# Functionalized metallic nanomaterials, nanozymes and nanomotors for emerging tumor diagnosis and treatment: from design to theranostics strategies

**DOI:** 10.7150/thno.131873

**Published:** 2026-05-01

**Authors:** Ze Wang, Dongzhou Wang, Bingya Zhang, Tong Sha, Di You, Mikhail I. Voevoda, Guokun Zhang, Bai Yang, Wenlai Guo, Quan Lin, Wenrui Qu

**Affiliations:** 1 Department of Hand Surgery, the Second Hospital of Jilin University, Changchun 130041, China.; 2 State Key Laboratory of Supramolecular Structure and Materials, College of Chemistry, Jilin University, Changchun, 130012, China.; 3 Department of Radiation Oncology, the Second Hospital of Jilin University, Changchun 130041, China.; 4 Department of Ultrasound Medicine, the Second Hospital of Jilin University, Changchun 130041, China.; 5 Department of Oral Pathology, Hospital of Stomatology, Jilin University, Changchun, 130021, China.; 6 Department of anesthesiology, China-Japan Union Hospital of Jilin University, Changchun 130033, China.; 7 Joint International Research Laboratory of Ageing Active Strategy and Bionic Health in Northeast Asia of Ministry of Education, Changchun 130041, China.; 8 Research Institute of Internal and Preventive Medicine-Branch of Institute of Cytology and Genetics SB RAS, Novosibirsk 630089, Russia.; 9 Institute of Antler Science and Product Technology, Changchun Sci-Tech University, Changchun, China.

**Keywords:** metallic nanomaterials, nanozymes, nanomotors, tumor, integration of diagnosis and treatment

## Abstract

Cancer is one of the most significant health issues and is the second leading cause of death worldwide. Traditional cancer diagnosis and treatment has serious defects and often fails to provide satisfactory results. Metallic nanomaterials, particularly gold-based nanomaterials, have been rapidly developed in the biomedical field, especially in the tumor theranostics owing to their unique morphological structures, outstanding physical and chemical properties, great biocompatibility and stability. With the development of research, researchers have introduced a second metal on the basis of single metallic nanomaterials to obtain bimetallic nanomaterials with better optical, catalytic and stability properties than single metallic nanomaterials. Based on the advantages of the above-mentioned metallic nanomaterials, many emerging types of metal-based materials have been developed accordingly. A typical example is metal-based nanozyme, which has inherent enzyme-like capabilities and has advantages such as great stability, abundant sources, controllable activity and low-cost preparation process compared with natural enzymes. The limited penetration of nanomaterials into tumor tissues is a major challenge in current cancer diagnosis and treatment. Further, another typical example is the metal-based nanomotor, which features a unique Janus structure and superior mobility, and is expected to enhance the deep penetration of nanomaterials into tumor, thereby improving the therapeutic effect of diseases. This review highlights the synthesis and properties of gold-based nanomaterials and bimetallic nanomaterials, with a focus on recent advances and future expectation in tumor diagnosis and treatment applications. With further research, we believe that the transition from metallic nanoparticles to metal nanozymes and then to metal enzymes-driven nanomotors will become important nanoplatforms for personalized cancer theranostics.

## 1. Introduction

With the continuous development of nanotechnology, metallic nanomaterials have emerged as a novel class of biomaterials and are currently the subject of extensive research. Metallic nanomaterials (such as Au, Pt, Ag, Cu, Mn, etc.) have shown great potential in biomedical applications, especially in the diagnosis and treatment of cancer, due to their unique nanoscale morphology, excellent physical and chemical properties, biocompatibility and good chemical stability 1.

Gold-based nanomaterials have excellent optical, electrical, magnetic, thermal and catalytic properties, and are extensively utilized across various fields, including physics, chemistry, catalysis, and biomedicine 2. In particular, gold-based nanomaterials represent one of the most studied metal-based nanomaterials in the field of nanomedicine. Based on the advantages of gold nanoparticles (AuNPs), such as exceptional physicochemical properties, facile synthesis, tunable size, favorable biocompatibility, and readily modifiable surface functionality, AuNPs are extensively utilized in biomedicine, particularly for tumor diagnosis and treatment.

Owing to their exceptional optical properties and high X-ray attenuation coefficient, AuNPs are highly suitable for fluorescence (FL) imaging and computed tomography (CT) imaging 3, 4. In addition, their excellent photothermal conversion capability enables them to serve as effective photothermal agents that convert near-infrared (NIR) light into heat for tumor photothermal therapy (PTT). Gold-based nanomaterials, as high-Z materials, exhibit strong attenuation of X-ray, enabling them to function as effective radiosensitizers to deposit radiation energy inside tumors as well as thereby enhance the effect of tumor radiotherapy (RT). In addition, the combination of thiols and amines on the AuNPs surface also provides a convenient way to introduce active functional groups. The introduction of the functional groups could endow AuNPs more functions, and could be used for labeling, targeting and intelligent drug release 5.

**Scheme 1:**
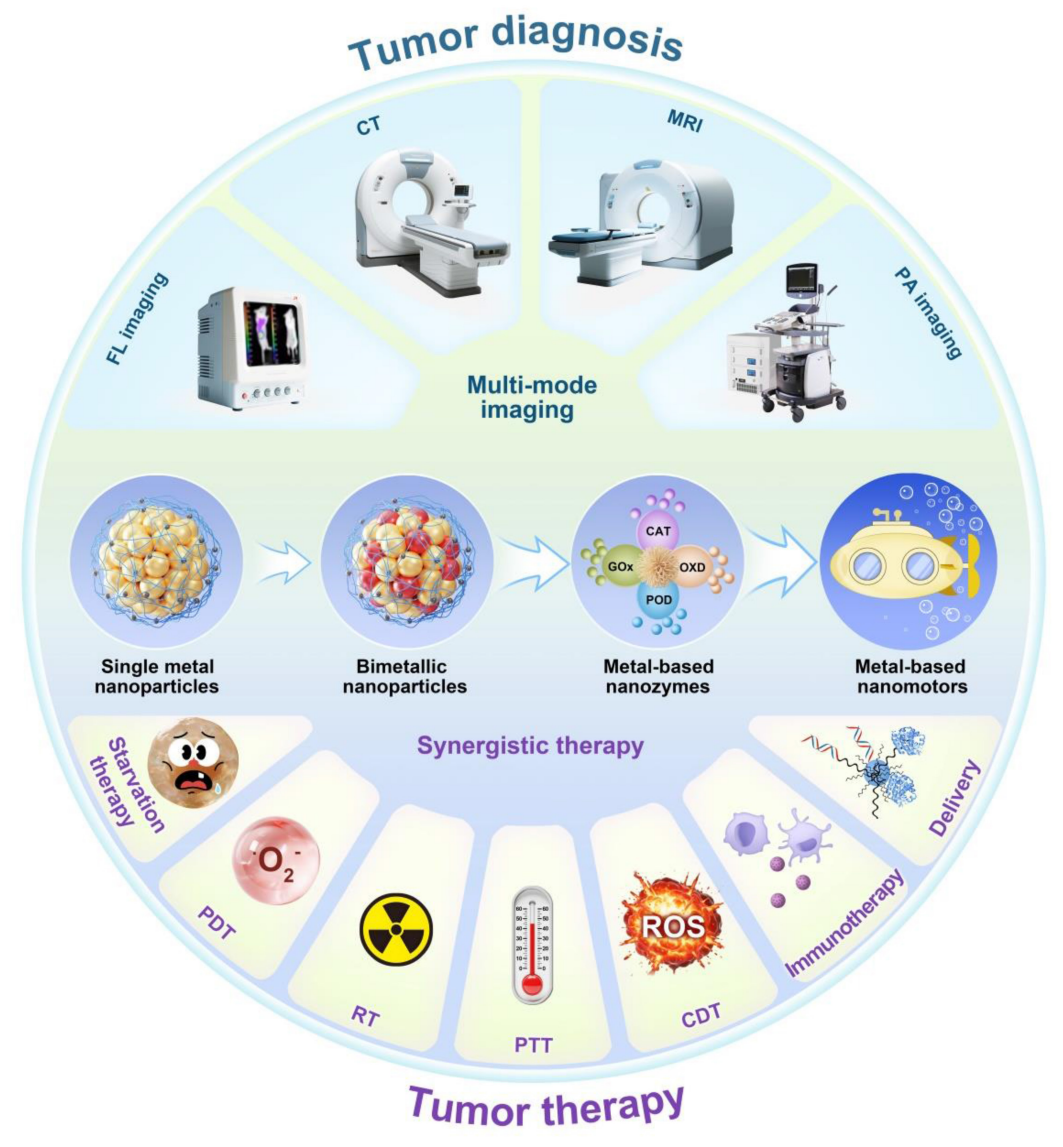
Functionalized metal-based nanomaterials, nanozymes and nanomotors for next-generation tumor diagnosis and treatment.

With the deepening of the research, the researchers introduced a second metal on the basis of single metallic nanomaterials to obtain a bimetallic nanomaterial **(Scheme 1)**. Studies have demonstrated that bimetallic nanomaterials exhibit comparable or superior physical and chemical properties compared to single metallic nanomaterials, and their great fluorescence, stability, enzyme-like activity and multi-mode imaging function, make them particularly well-suited for tumor diagnosis and treatment 6. For example, the introduction of transition metals (such as Cu, Fe, Mn, etc.) on the basis of AuNPs can endow them catalytic activity and diversified enzyme-like functions to achieve more effective tumor therapy. Incorporating Ag into AuNPs significantly enhances fluorescence emission intensity and quantum yield, resulting in better tumor FL imaging 7. By introducing magnetic metals such as Gd or Mn on the basis of AuNPs, the magnetic resonance imaging (MRI) function can be obtained on the basis of the FL and CT imaging function of the original AuNPs. Therefore, bimetallic nanomaterials to a certain extent solve the limitations of single-metal nanomaterials mediated cancer theranostics.

Based on the advantages of the above-mentioned metallic nanomaterials, many new types of metal-based materials have been developed successively **(Scheme 1)**. Metal-based nanozymes are a typical example. They have inherent enzyme-like capabilities. Compared with natural enzymes, they have the advantages of stability, abundant sources, controllable activity, and low preparation process cost 8. However, the limited penetration of nanomaterials into tumor tissues is the main challenge in current cancer treatment 9. Therefore, another typical example is the metal-based nanomotor, which features a unique Janus structure and superior mobility, and is expected to enhance the penetration of nanomaterials into tumor, improving therapeutic effect of cancer 10, 11.

Recognized as one of the most serious public health concerns worldwide, cancer has greatly threatened the health of people all over the world. Traditional treatments often produce serious side effects and unsatisfactory results. Following successive breakthroughs in nanoscale fabrication and functionalization, gold-based nanomaterials and bimetallic nanomaterials, as effective nanomedicines, have made great progress in the diagnosis and treatment of diseases. This review presents an overview of the synthesis and properties of gold-based and bimetallic nanomaterials, highlighting their recent applications in tumor diagnosis and treatment, along with a brief discussion of existing challenges and future directions, in order to offer strategic guidance for further research.

## 2. Metallic nanomaterials

### 2.1. Gold-based nanomaterials

Gold-based nanomaterials are one of the most widely studied subjects in nanoscience and nanotechnology, and also one of the most studied metal-based nanomaterials in nanomedicine. Gold-based nanomaterials have excellent optical, electrical, magnetic, thermal and catalytic properties, and have been widely used in physics, chemistry, catalysis and biomedicine fields 2, 12. Gold-based nanomaterials have garnered significant research attention owing to their facile synthesis and unique physicochemical properties. At present, diverse synthetic methodologies have been developed for preparing gold nanoparticles (AuNPs), encompassing physical, chemical, biological 13. We conducted a comprehensive comparative analysis of the synthesis methods for gold nanoparticles, as shown in **Table 1**.

Common physical methods include γ-ray irradiation, ultraviolet irradiation and laser irradiation 14. AuNPs prepared using γ-ray irradiation have controllable size and high purity 15. At the same time, AuNPs with controlled size can be prepared using ultraviolet irradiation method too. Different wavelengths of ultraviolet irradiation are adopted to promote chemical reactions in solutions of Au ions 16. Laser irradiation method makes use of the photoinduced effect of a laser beam (a wavelength of 532 nm) to reduce chloroauric acid (HAuCl_4_), thereby enabling AuNPs (<5 nm) to be prepared 17. This is an effective physical method to prepare AuNPs with controllable features, providing accurate and repeatable characteristics.

Commonly employed methods in chemical synthesis include reduction and electrochemical reduction. The chemical synthesis of AuNPs typically entails the reduction of HAuCl₄ solution in the presence of the stabilizing agent. A diverse range of reducing agents are frequently utilized for this purpose, such as citrate, sodium borohydride, and hydrazine hydrate. AuNPs are usually synthesized electrochemically using a simple two-electrode cell 18. The particle size is regulated by adjusting the surfactant concentration, growth temperature as well as current density. As a result, AuNPs synthesized by electrochemical method has the advantages of superior quality, fast synthesis speed and easy to control the yield.

Biological methods provide the clean, non-toxic, as well as environmentally friendly approach for synthesizing AuNPs, employing plant-derived compounds, bacteria, algae, yeast, and viruses. Bharadwaj *et al*. summarized plant-based methods for the preparation of AuNPs and discussed their physicochemical properties 19. They also discussed the latest breakthroughs and results of green synthetic AuNPs in tumor therapy. In addition, it has been found that microorganisms such as yeast, bacteria and algae can adsorb and accumulate metals 20, secrete enzymes to hydrolyze metals, and make metal ions undergo enzymatic reduction, thus improving the reduction rate of metal ions 21.

**Figure 1:**
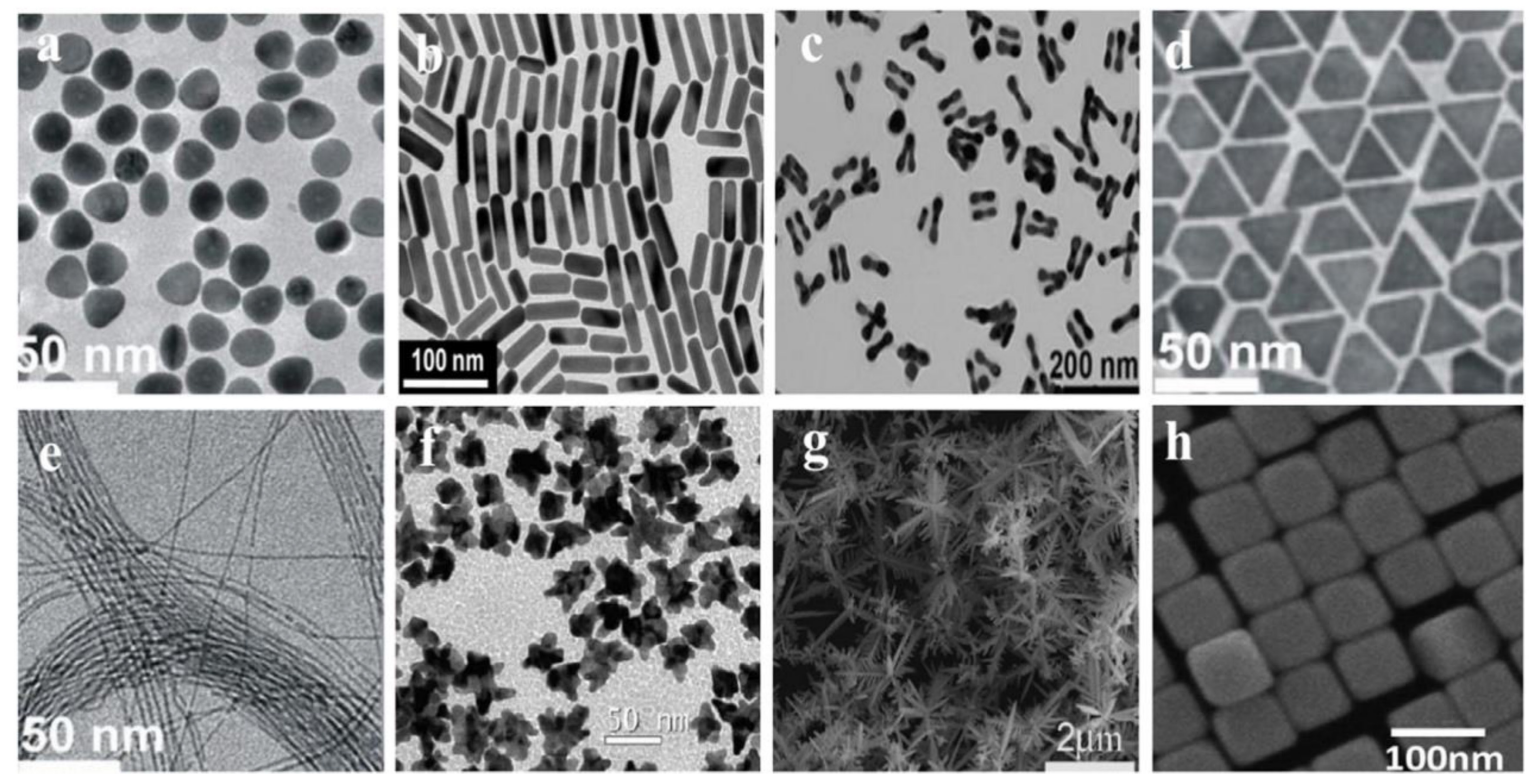
Common morphologies of gold-based nanomaterials. (a-f) TEM images of gold nanoparticles: (a) quasi-spheres, (b) nanorods, (c) nanodumbbells, (d) triangular nanoprisms, (e) ultrathin nanowires, (f) nanostars; (g-h) SEM images of gold nanoparticles: (g) nanodendrites, (h) nanocubes. Adapted with permission from 17. Copyright © 2018 Elsevier B.V. All rights reserved.

The size, shape and morphology of gold-based nanomaterials play a critical role in determining their properties and subsequent applications. Consequently, recent research focused on developing synthesis methods for gold-based nanomaterials with different morphologies and sizes. Common morphologies of gold-based nanomaterials include nanospheres, nanorods, nanowires, nanocages, and nanowires** (Figure 1)**.

Nanospheres are the most common morphologies of gold-based nanomaterials. To date, researchers have developed many synthetic strategies to synthesize gold nanospheres with high dispersion and a wide size range. In general, in the presence of a reducing agent, reducing HAuCl_4_ solution readily yields nanospheres with diameters of 2-100 nm 13. At present, there are different methods to synthesize gold nanospheres. Wang *et al*. summarized synthetic methods for gold nanospheres using protective ligands, including biomolecules, surfactants, polymers, and dendrimers 22. Gold nanorods (AuNRs) are one of the most widely used anisotropic nanoparticles. In general, AuNRs with a controlled aspect ratio are prepared by seed-growth and electrochemical methods 23. Gold nanowires with a high aspect ratio (L/D > 500) are considered to be the cornerstone of nanostructure-based sensor components in the field of electrochemistry due to the high surface-volume ratio, anisotropy and self-assembly capability 24. Many methods have been developed for synthesizing of gold nanowires, such as template-assisted growth and seed-mediated growth 25. Gold nanocages are hollow, porous AuNPs with sizes ranging from 10-150 nm. Gold nanocages have been widely used in the field of electrochemistry 26. Gold nanocages can be mass-produced by a simple electrical substitution reaction, which typically occurs between HAuCl_4_ and silver nanostructures (such as nanocubs and nanospheres). By adjusting molar ratio of Ag to HAuCl_4_, the performance of the gold nanocages can be easily controlled. For example, Chen *et al*. synthesized gold nanocages through an electrical substitution reaction between HAuCl_4_ and Ag nanocubs 27. Raveendran *et al*. established a microwave-assisted approach for fabricating gold nanocages with enhanced simplicity 28, enabling accelerated synthesis under benign conditions with scalable output.

Gold nanoparticles with virus-like spikes (AuNVs) are superior to gold nanospheres and nanostars in terms of cellular uptake, transcellular transport efficiency, and tumor penetration depth, highlighting the critical role of nanoparticle morphology and size in determining tumor theranostic efficacy 29. As a result, they achieve more effective chemophotothermal therapy and inhibit the growth of colorectal cancer. In addition, studies have shown that gold nanorods have better tumor targeting and accumulation, higher drug loading and release, longer blood half-life, and better tumor inhibition than gold nanospheres. This is because the rod-like structure has a larger specific surface area, which improves the drug loading efficiency. Moreover, it diffused faster and penetrated deeper in the tumor stroma. The rod structure can escape the phagocytic clearance of the blood and reticuloendothelial system, prolong the blood circulation time, and increase the chance of penetration of the tumor site 30. The morphology of gold nanomaterials significantly influences tumor radiotherapy efficacy. Ma *et al*. synthesized three morphologically distinct but size-matched (~50 nm) gold nanostructures: spherical nanoparticles (GNPs), nanospikes (GNSs), and nanorods (GNRs). Under 4 Gy X-ray irradiation, their sensitization enhancement ratios (SERs) were 1.62, 1.37, and 1.21 respectively, demonstrating GNPs' superior radiosensitization effect 31. Crucially, the antitumor activity of gold nanomaterials also has a size dependence. In comparison, smaller AuNPs (< 20 nm) have better fluorescence emission, better tissue penetration, depth and better tumor homogeneity. However, these smaller AuNPs also present with quick renal excretion, which leads to the poor accumulation in tumours and affected diagnostic and therapeutic efficiency 32. Although AuNPs larger than 100 nm can act as efficient photothermal agents by the enhanced permeability and retention (EPR) effect and could present strong absorption of near-infrared (NIR) light. However, smaller AuNPs are sometimes captured by reticuloendothelial system and limit the therapeutic effects 33.

Along with the excellent protective effect on AuNPs, surface ligand design also involves the synthesis of gold-based nanomaterials. Besides serving as the outer layer of AuNPs, surface ligands have a two-function regulation property: (1) As the outer shell of AuNPs, they directly impact the relationship between AuNPs and the external environment (such as solvents, molecules and cells, etc.) in different applications. (2) The interface chemistry of ligands to gold atom affects structure and the physicochemical properties of gold-based nanomaterials. Especially, the ligand (especially the electron donating atoms/groups) enriched ligand can further enhance the ligand-to-metal-core charge transfer which will raise the luminescent effect of AuNPs. Selecting appropriate ligands with relatively strong molecule physicochemical properties can protect the gold-based nanomaterials better and improve the stability of the gold-based nanomaterials in solution. Consequently, ligand selection strategies decide the function performance of gold-based nanomaterials for these applications 34. The choice of ligands for AuNPs need to be evaluated on three main aspects: (1) covalent bonding at ligand–gold level (e.g. Au-S, Au-P); (2) intermolecular physical forces (e.g. hydrophobic force, van der Waals force); (3) ionic interaction functionated groups (e.g. carboxyl group, amine group) 35.

**Figure 2:**
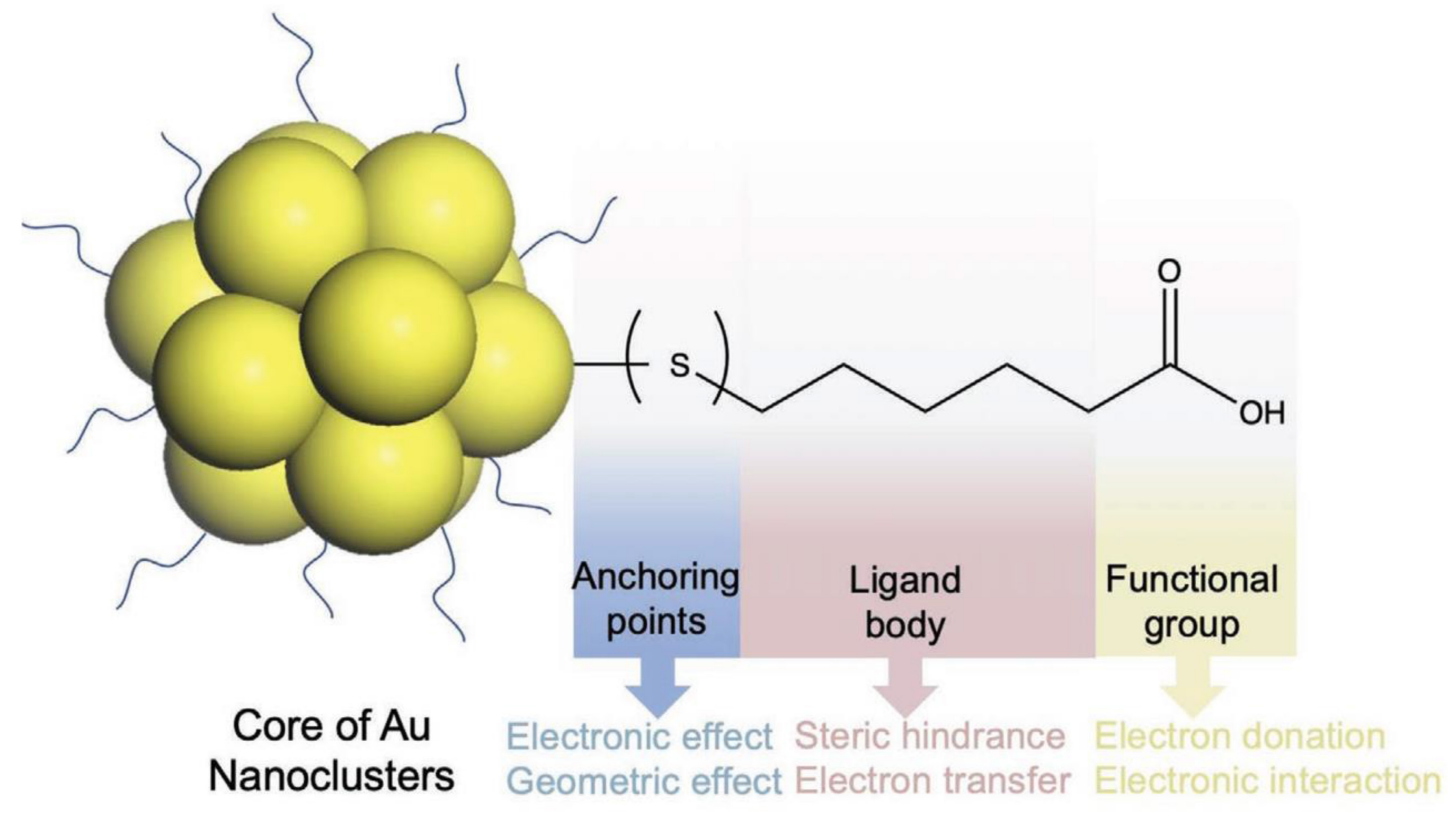
Schematic diagram of the protective ligand on AuNPs, with mercaptohexanoic acid as the model. Adapted with permission from 35. Copyright © 2021 Wiley-VCH GmbH.

The organic ligands on the AuNPs surface are composed of three parts, as shown in **Figure 2**. The anchoring group, interacting directly with gold atoms (Au(I) or Au(0)), constitutes the innermost ligand component. These groups-typically sulfur, selenium, phosphorus, or carbon-form strong covalent bonds with surface Au atoms. Hence, the choice of anchoring points dictates the structure and physicochemical properties of gold-based nanomaterials. The spacer unit (middle segment) of surface ligands, such as alkyl chains of varying lengths or substituted/unsubstituted benzene rings, provides conjugative capacity. Additionally, intermolecular physical forces (e.g., hydrophobic interactions and van der Waals forces) between adjacent ligands affect AuNPs properties through steric hindrance and ligand-to-metal electron transfer mechanisms 36. The third part is the functional groups on ligands (mainly applicable to hydrophilic ligands), such as carboxyl as well as amine groups 37, 38. The functional group on hydrophilic AuNPs critically govern their solubility and application performance, as ligand composition will affect the interaction of AuNPs with the external environment, including solvents, molecules, cells as well as tissues 39. In summary, all three parts synergistically determine the physicochemical properties of AuNPs, functioning as an integrated molecular architecture.

**Figure 3:**
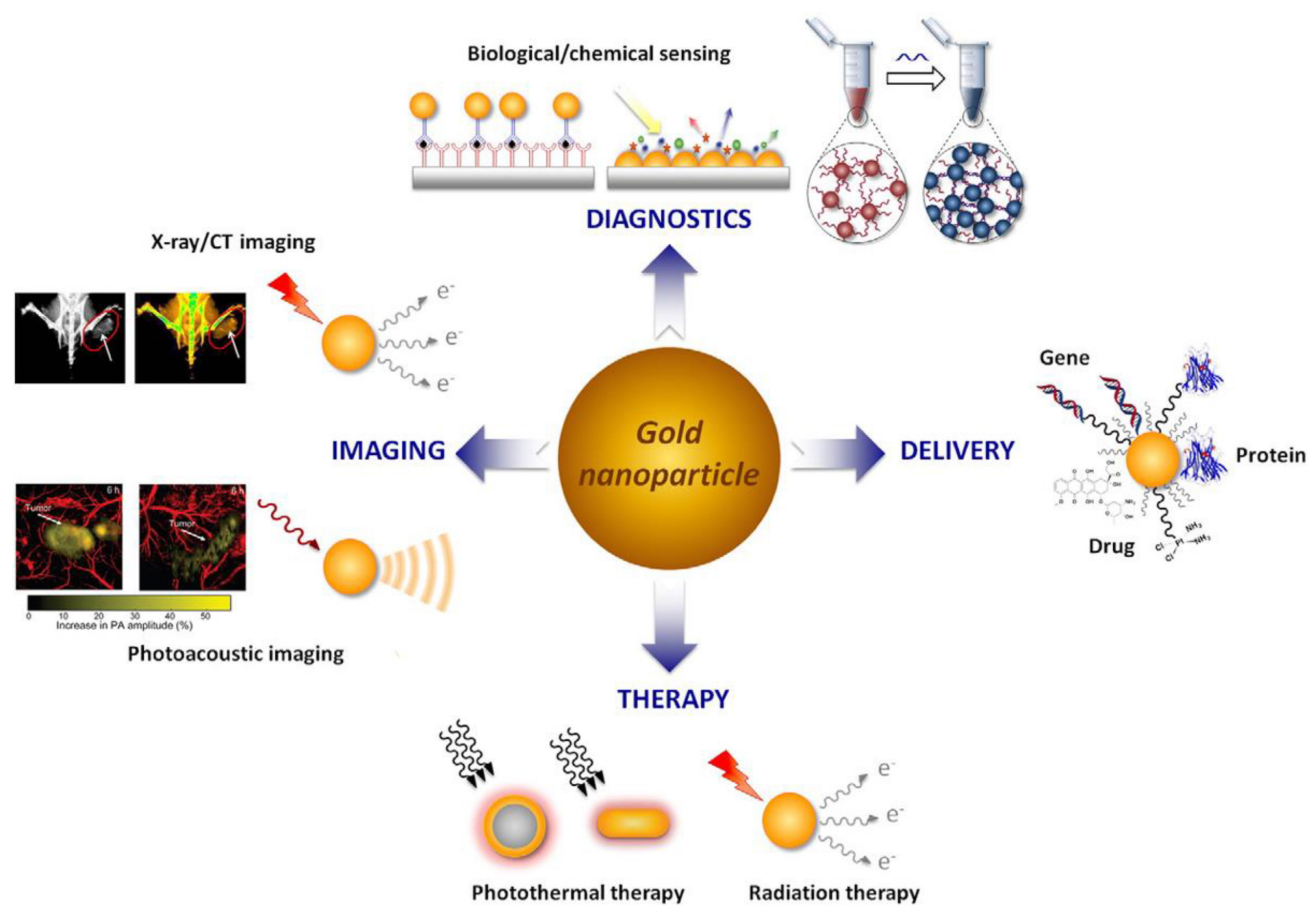
Biomedical applications of AuNPs. Adapted with permission from 5. Copyright © 2015 Elsevier B.V. All rights reserved.

Based on the advantages of AuNPs, such as excellent physical and chemical properties, good biocompatibility, adjustable size and easy surface functionalization, AuNPs are extensively employed in biomedical applications **(Figure 3)**. AuNPs have been reported to have good optical properties and high X-ray attenuation coefficient, enabling its broad application in FL and CT imaging 4, 40. AuNPs have excellent photothermal conversion ability, consequently, AuNPs serve as an exceptional photothermal agent, converting NIR radiation into thermal energy for PTT. Gold-based nanomaterials, as high-Z materials, have a strong attenuation ability to X-ray, functioning as excellent radiosensitizers, that selectively deposit radiation energy within tumors to enhance RT efficacy 41. Additionally, the combination of thiols and amines on the AuNPs surface also provides a convenient way to introduce reactive functional group. The introduction of the functional group could endow AuNPs more functions that could be used for labeling, targeting, and intelligent drug release 5.

### 2.2. Other metallic nanomaterials

#### 2.2.1. Silver-based nanomaterials

Silver-based nanomaterials represent another extensively investigated class of metallic nanomaterials in nanomedicine. Silver nanoparticles (AgNPs) are widely utilized across biomedical and food technology, owing to their physical, chemical as well as biological properties 42.

**Figure 4:**
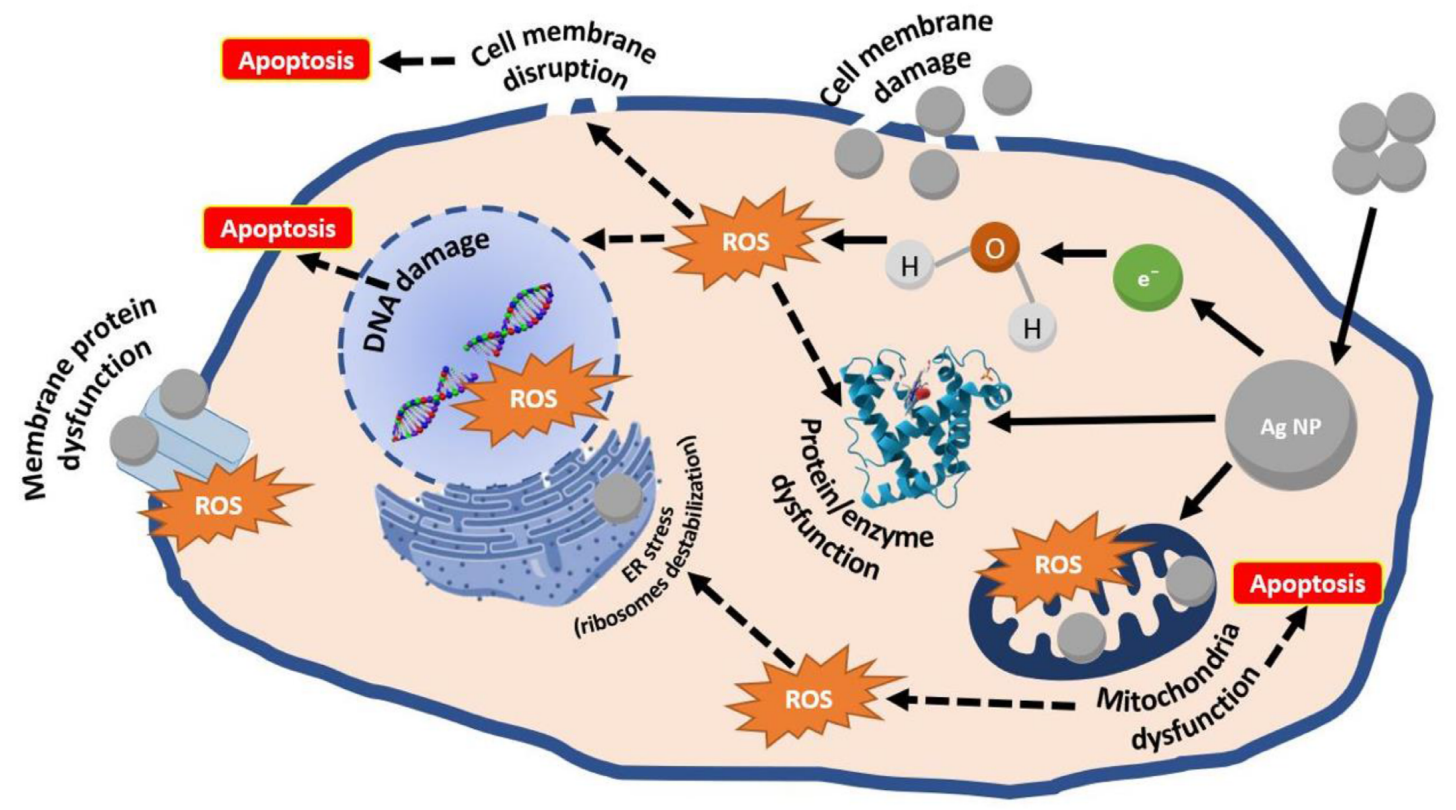
Schematic representation of AgNPs anticancer mechanism. Adapted with permission from 43. Copyright © 2022 by the authors. Licensee MDPI, Basel, Switzerland.

Recently, the inherent anticancer activity of AgNPs has prompted growing research interest in their application for tumor diagnostics and therapeutics. AgNPs exert their antitumor effects by disrupting cell membrane fluidity and inhibiting uncontrolled proliferation. Mechanistically, they release Ag^+^ that capture electrons, thereby enhancing intracellular oxidative stress, elevating reactive oxygen species (ROS) levels, and depleting adenosine triphosphate (ATP), ultimately suppressing cancer cell proliferation. Studies have shown that Ag^+^ release occurs predominantly in mitochondria and secondarily in the nucleus, where they interact with DNA, inducing double-strand breaks and subsequent cell death. Furthermore, AgNPs can induce early apoptosis in a p53-independent manner and modulate cancer cell autophagy. The above AgNPs mechanism of action is summarized in **Figure 4**.

#### 2.2.2. Copper-based nanomaterials

Benefiting from inherent physicochemical and biological properties, along with essential roles in living organisms, copper-based nanomaterials have emerged as promising platforms for cancer theranostics, with applications spanning chemodynamic therapy (CDT), photothermal therapy (PTT), photodynamic therapy (PDT), and drug delivery. Cu (I) can promote ROS production through Fenton-like reaction. Studies confirm that Fenton-like reactions catalyzed by Cu(I) exhibit superior kinetics to those mediated by Fe(II), with a rate constant of 1×10^4^ M^-1^s^-1^, approximately 160-fold higher than Fe(II)'s activity, even under neutral or weakly acidic conditions 44. Therefore, many copper-based nanomaterials have been developed for CDT in cancer. Concurrently, copper-based nanomaterials demonstrate promising PTT performance, attributed to their minimal cytotoxicity, cost-effectiveness, and strong NIR absorption 45. In general, the localized surface plasmon resonance (LSPR) effect of noble metals is generated by free electron oscillations. The difference is that the LSPR of copper-sulfur materials is generated by free electrons oscillation 46. Li *et al*. prepared 5.6-nm CuS nanodots as multifunctional photothermal agents 47. They irradiated 100 μg/mL CuS nanodots with the near-infrared laser (808 nm, 2 W/cm^2^) for 10 minutes, achieving a temperature elevation of 27 °C, showing excellent PTT capability. In addition, the size of CuS nanodots is small, and its spleen uptake is only 24% and liver uptake is 15%. CuS nanodots have good photothermal therapeutic effect and renal clearance, which exhibit strong viability for clinical implementation. In addition, copper-based nanoagents are engineered as responsive drug carriers due to the excellent biocompatibility, high surface-to-volume ratio, exceptional stability, as well as photoresponsive properties 48. In tumor imaging, copper-based nanomaterials also play an important role, such as positron emission tomography (PET), photoacoustic imaging (PAI), and MRI.

#### 2.2.3. Manganese-based nanomaterials

**Figure 5:**
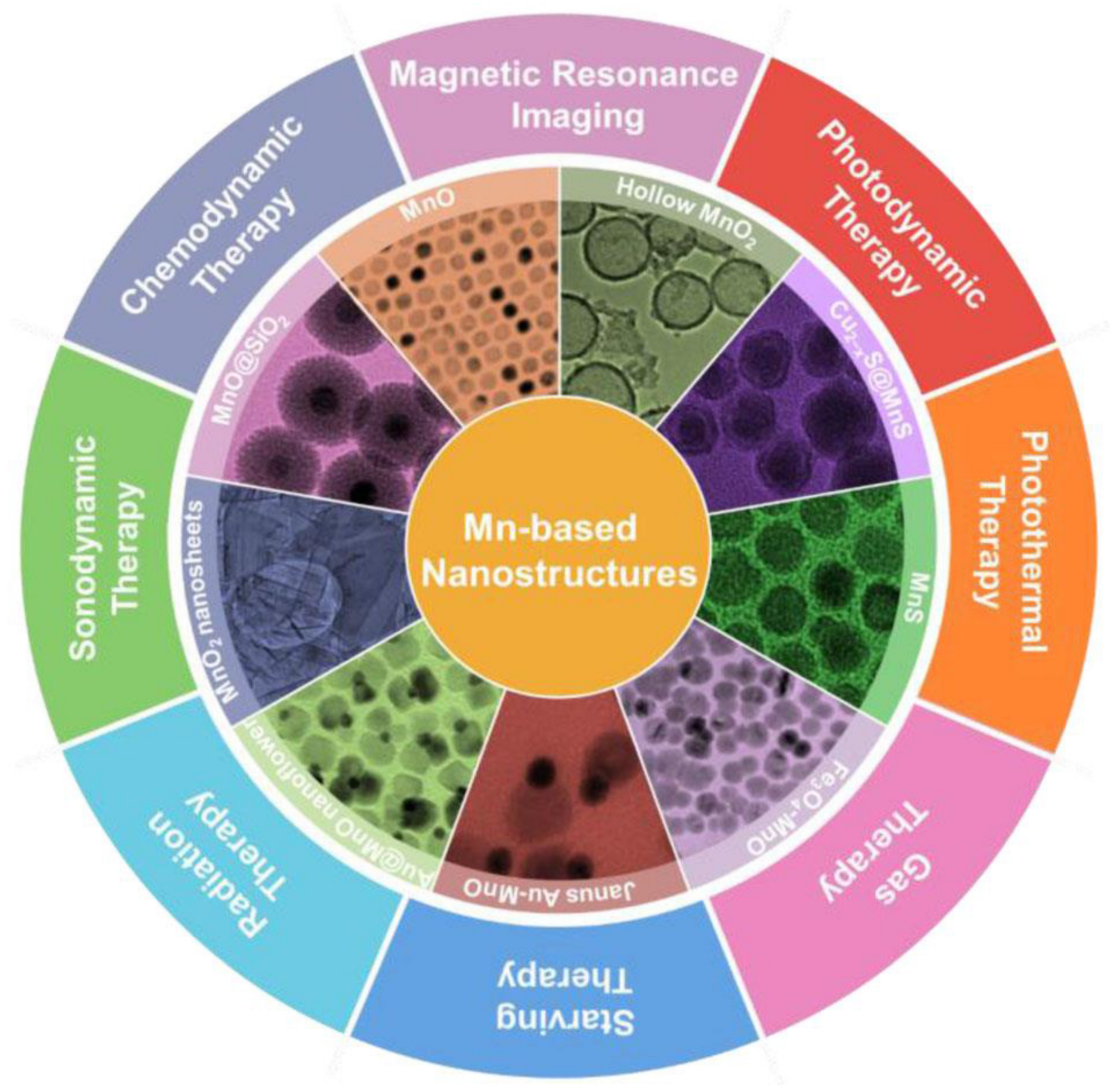
Schematic illustration of potential applications of Mn-based nanostructures. Adapted with permission from 49. Copyright © 2021 Wiley-VCH GmbH.

Research on manganese-based nanomaterials in biological imaging and cancer treatment have aroused considerable interest. This is largely attributed to their unique characteristics, including tunable morphological and structural features, novel magneto-optical behavior, strong catalytic activity and good biodegradability. To date, researchers have synthesized diverse classes of manganese-based oxides, sulfides and the hybrid nanostructures and used them in cancer diagnosis and therapy **(Figure 5)**. Manganese-based nanomaterials have been used in tumor therapy, including PDT, sonodynamic therapy (SDT), RT, CDT, ferroptosis-mediated therapy, gene therapy, gas therapy and PTT. Manganese-based nanoparticles can mimic the catalytic function and activity of some biological enzymes on tumor microenvironment (TME) 49. Manganese ions (II) can mimic horseradish peroxidase and induce the highly expressed hydrogen peroxide (H_2_O_2_) in TME to produce ROS through Fenton-like reaction, thereby producing specific cytotoxicity at the tumor site and achieving CDT 50. Mangane-based nanomaterials can also mimic catalase (CAT), catalytically decomposing hydrogen peroxide into water and O₂ to alleviate tumor hypoxia. Furthermore, mangane-based nanomaterials consume overexpressed glutathione (GSH) in the TME through redox reactions, which can prevent the scavenging of ROS generated by Fenton-like reaction 51.

In addition, manganese (II) ion exhibit excellent paramagnetic properties characterized by high spin number (S=5/2, I=5/2), prolonged electron spin relaxation and 5 unpaired electrons 49. Higher oxidation states of manganese ions correlate with reduced unpaired electron counts and diminished T_1_ relaxivity. Consequently, the development of manganese-based T_1_-weighted MRI contrast agents primarily targets Mn^2+^ species. As established paramagnetic metal agents, Mn^2+^-based materials have received clinical approval for intravenous administration, exemplified by hepatic imaging agent MnDPDP and oral contrast agent manganese chloride 52, 53.

### 2.3. Bimetallic Nanomaterials

**Figure 6:**
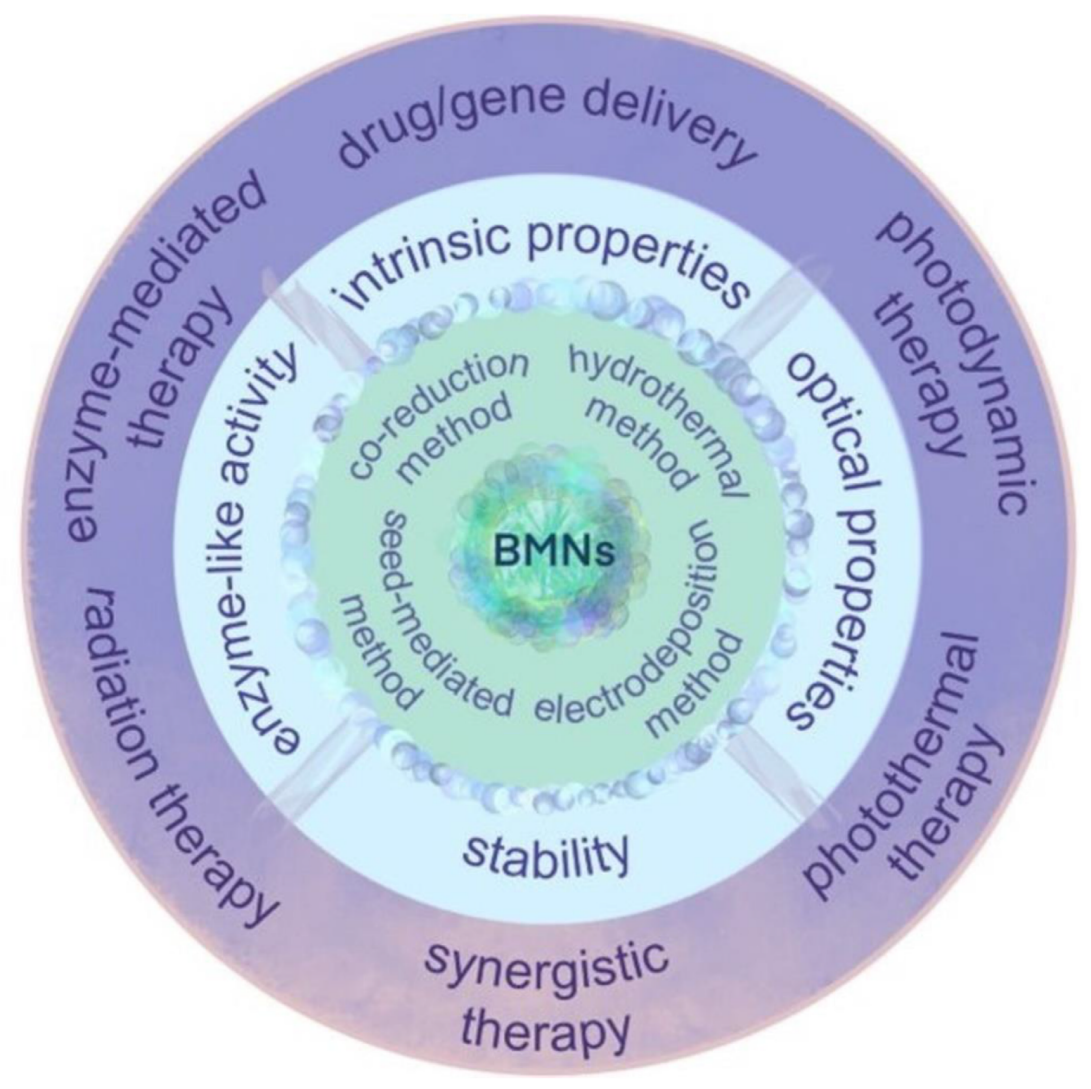
Synthesis methods, unique properties and application of bimetallic nanomaterials (BMNs) in cancer therapy. Adapted with permission from 58. Copyright © 2022 by the authors. Licensee MDPI, Basel, Switzerland.

In actual tumor diagnosis and treatment, single-metal nanomaterials suffer from problems such as low fluorescence property and photothermal conversion efficiency, single catalytic/enzymatic activity, poor stability, and limited tumor diagnosis and treatment functions. Bimetallic nanomaterials are bimetallic nanoalloys, intermetallics and composites composed of two different metal nano-molecules. Bimetallic nanomaterials are composed of two different metal elements, mixed in specific pattern and structure; their physical and chemical properties are better than those of the monometallic ones. By virtue of their specific morphologies, physical and chemical properties, good biocompatibility and the synergy effect, bimetallic nanomaterials have been explored for biomedical uses, especially for cancer **(Figure 6)**.

Bimetallic nanomaterials have been shown to possess physicochemical properties comparable to or even surpassing those of single metallic nanomaterials. The exceptional fluorescence, photothermal performance, photocatalytic activity, and enzyme-like characteristics of these materials significantly enhance their applicability in tumor theranostics 6, 54. Therefore, bimetallic nanomaterials have garnered significant attention in the medical research community. Particularly, those exhibiting SPR effects have enabled advanced cancer therapy owing to their superior photothermal conversion capabilities. In addition, compared with single metallic nanomaterials, bimetallic nanomaterials have great photothermal stability as well as can adjust the photothermal conversion efficiency more accurately, which solve the problem of low PTT efficiency of single metallic nanomaterials 55. Simultaneously, bimetallic nanomaterials leverage their unique physicochemical properties, particularly surface chemistry to exhibit enhanced biocompatibility, drug-loading capacity, and radiosensitization efficacy. These advancements effectively address limitations inherent to single-metallic nanomaterial-mediated cancer therapy 56, 57.

#### 2.3.1. Synthesis method of bimetallic nanomaterials

**Figure 7:**
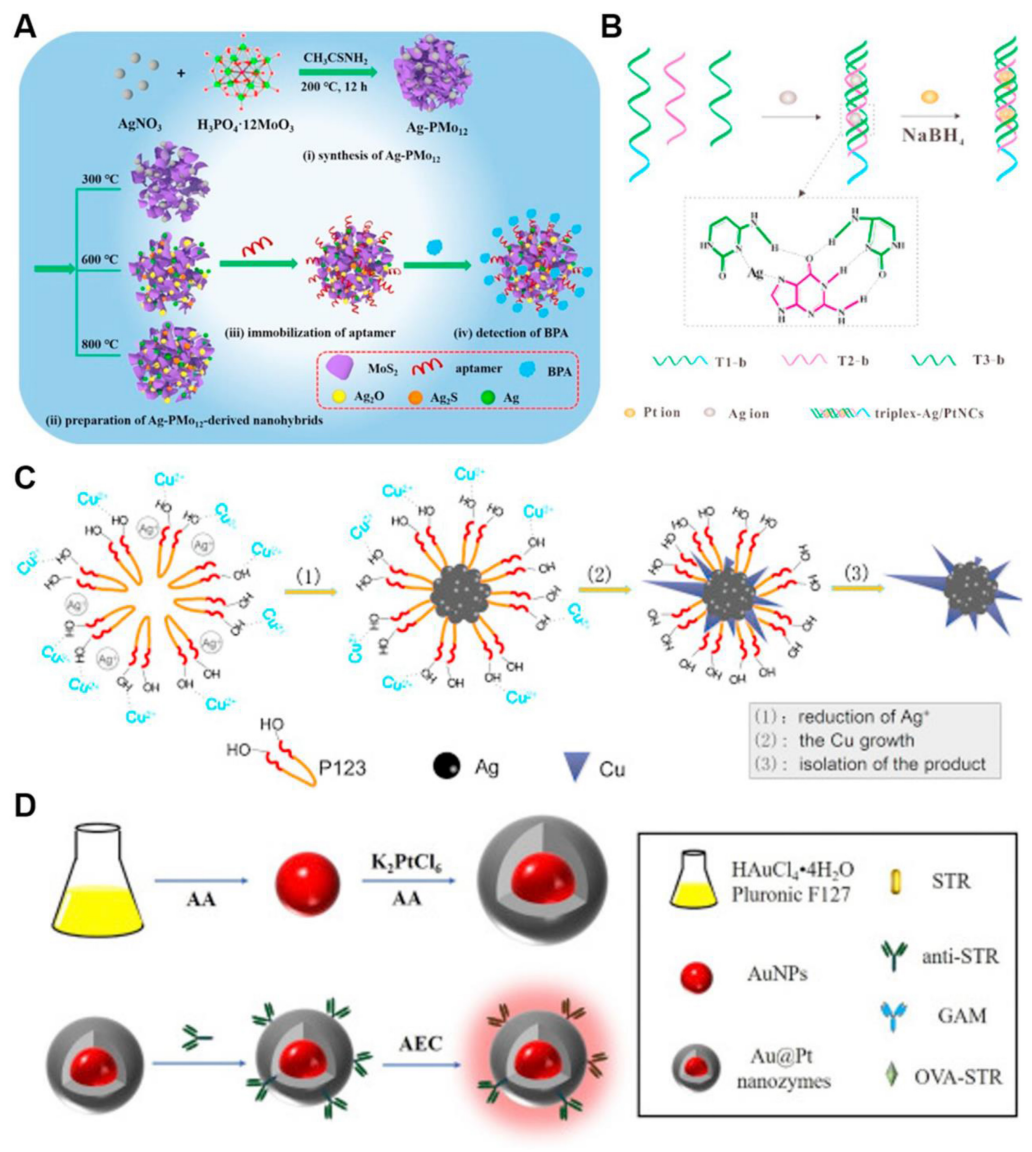
Different approaches for preparing bimetallic nanomaterials. (a) Fabrication of AgMo mesoporous nanosheets for electrochemical detection of BPA; (b) Formation mechanism of AgPt nanoclusters; (c) Structural representation of dendritic bimetallic AgCu nanomaterials; (d) Schematic diagram of preparing AuPt core-shell. Adapted with permission from 60. Copyright © 2020 Elsevier B.V.

Many synthesis methods of bimetallic nanomaterials have been reported, such as hydrothermal, co-reduction, seed-mediated growth as well as biological method. Liu *et al*. reviewed controlled synthesis strategies for architecturally diverse bimetallic nanomaterials, encompassing crown-jewel, hollow, heterostructured, core-shell, alloyed, and mesoporous frameworks 59. **Figure 7** shows some examples of synthetic routes. Common bimetallic synthesis methods are described in detail below.

Hydrothermal synthesis is commonly employed for fabricating bimetallic nanomaterials. After heating, the decomposition and reduction of metal precursors are promoted. Hydrothermal method is suitable for reactions with low reduction potential and difficult direct reduction. Bimetallic nanomaterials with low reduction potential have been synthesized by hydrothermal methods, such as CoNi, NiRe and CuNi 61. Li *et al*. synthesized Cu/Co bimetallic nanomaterials by hydrothermal method and systematically investigated the chemiluminescence (CL) catalytic properties. The results showed that the strength of CL can be increased by 67.9±3.5% due to the strong synergistic catalysis of Cu and Co. Based on the phenomenon, a steric hindrance strategy for cancer cell detection was constructed 62. Mariyappan *et al*. fabricated a SmMoS bimetallic sulfide nanoflake architecture via a hydrothermal route, where layered MoS_2_ is wrapped with α-Sm_2_S_3_. This composite was employed as an electrochemical sensor for the antineoplastic drug 5-fluorouracil, demonstrating a low detection limit of 0.015 μM in clinical samples 63.

In a typical co-reduction (or one-pot) process, two metal precursors are mixed and reduced to yield bimetallic alloys or intermetallics 64, such as AuAg, AgCu, AgPt, PdAu, AuPd, AuPt, PdIr and FeCu. This straightforward strategy is favored for its operational ease, low cost, and short reaction times. Notably, the final morphology and crystal structure of these nanomaterials can be tailored by adjusting reaction temperature, surfactant and reducing agent selection, as well as ligand characteristics. Da Silva *et al*. prepared SiO_2_-supported AgAu bimetallic nanomaterials by co-reduction method using metal salts precursors. AgAu bimetallic nanomaterials are prepared by NaBH_4_ reduction after the introduction of AgNO_3_ and HAuCl_4_ based on amorphous SiO_2_ 65. Wang *et al*. prepared Au/CuNDs with an ultrasmall size and great biocompatibility were prepared using mercaptosylated polyethylenimine as ligands by co-reduction method. Following copper incorporation, the emission wavelength of Au/CuNDs red-shifted to the NIR-II region (1006 nm), establishing an optimal nanoprobe platform for high-resolution tumor imaging via near-infrared fluorescence 66.

Seed-mediated growth represents a widely adopted strategy for fabricating plasmonic noble metallic nanocrystals 67. Due to the good morphology, size and surface composition of the prepared nanocrystals, the method has been applied to prepare bimetallic nanomaterials. In general, seed-mediated growth method fundamentally enables the fabrication of anisotropic metal structures and core-shell structures. Zhan *et al*. prepared Pd-Cu bimetallic nanomaterial using seed-mediated co-reduction method, which is simple to operate and has controllable morphology.

Biological method is a new method for preparing bimetallic nanomaterials. This approach employs biological components-including foliar extracts, plant-derived metabolites, proteins, and DNA-as reductants or structural templates for controlled growth 60. Ramos *et al*. discussed green synthesis methods for bimetallic nanostructures and highlighted bio-derived agents (such as plant extracts, DNA, proteins) as a green ingredient for bimetallic nanostructures. They emphasized biosynthesis schemes leading to controllable nanoscale features of nanoparticles, such as size, composition, morphology and configuration. For instance, AgPt nanoclusters are synthesized with triplex DNA as template to obtain highly sensitive and biocompatible AgPt nanoclusters **(Figure 7b)**. Ma *et al*. prepared PdPt_3_ dendritic nanoparticles stabilised by lentinan (PdPt_3_-LNT NDs) with a biotemplating strategy. The obtained nanostructures display an intrinsic oxidase-mimicking behavior. Superoxide anions and singlet oxygen was determined to be the reactive molecules responsible for the activity of PdPt_3_-LNT NDs. 68. Yallappa *et al*. successfully synthesized eco-friendly FeNi bimetallic nanomaterial with high efficiency of antibacterial activity using Jasminum leaf extract as both reducing and stabilizing reagent 69. At the same time, this biosynthesis approach achieves eco-compatible synthesis under mild conditions by using biological components and prepares bimetallic nanomaterials to acquire excellent inherent biocompatibility.

#### 2.3.2. Optical properties of bimetallic nanomaterials

Single-metal nanomaterials have some disadvantages, such as poor fluorescence properties and low photothermal conversion rate. Therefore, introducing another metal to produce bimetallic nanomaterials and making optimal use of parameters such as metal ratio, metal size, metal morphology and the synergetic plasmonic effect can improve the optical properties of single-metal nanomaterials, and enhance their applicable scope for tumor theranostics. Bimetallic nanomaterials have characteristics of localized surface plasmon resonance (LSPR), endowing them with excellent optical properties. At the same time, bimetallic nanomaterials have been highly valued by researchers for near-infrared tunable plasmonic properties and higher chemical stability 70. LSPR of bimetallic nanomaterials (the coherent oscillation caused by the incident light of conduction electrons in bimetallic nanomaterials and light energy dissipation later on) promotes their varied applications in photothermal conversion, photocatalytic process and biosensor 71. Furthermore, structure and morphology decide how bimetallic nanomaterials respond to the light. While harnessing this structure–property relationship, precise engineering of their size and shape helps effectively regulate their optical performance and makes bimetallic nanomaterials have versatile applications.

**Figure 8:**
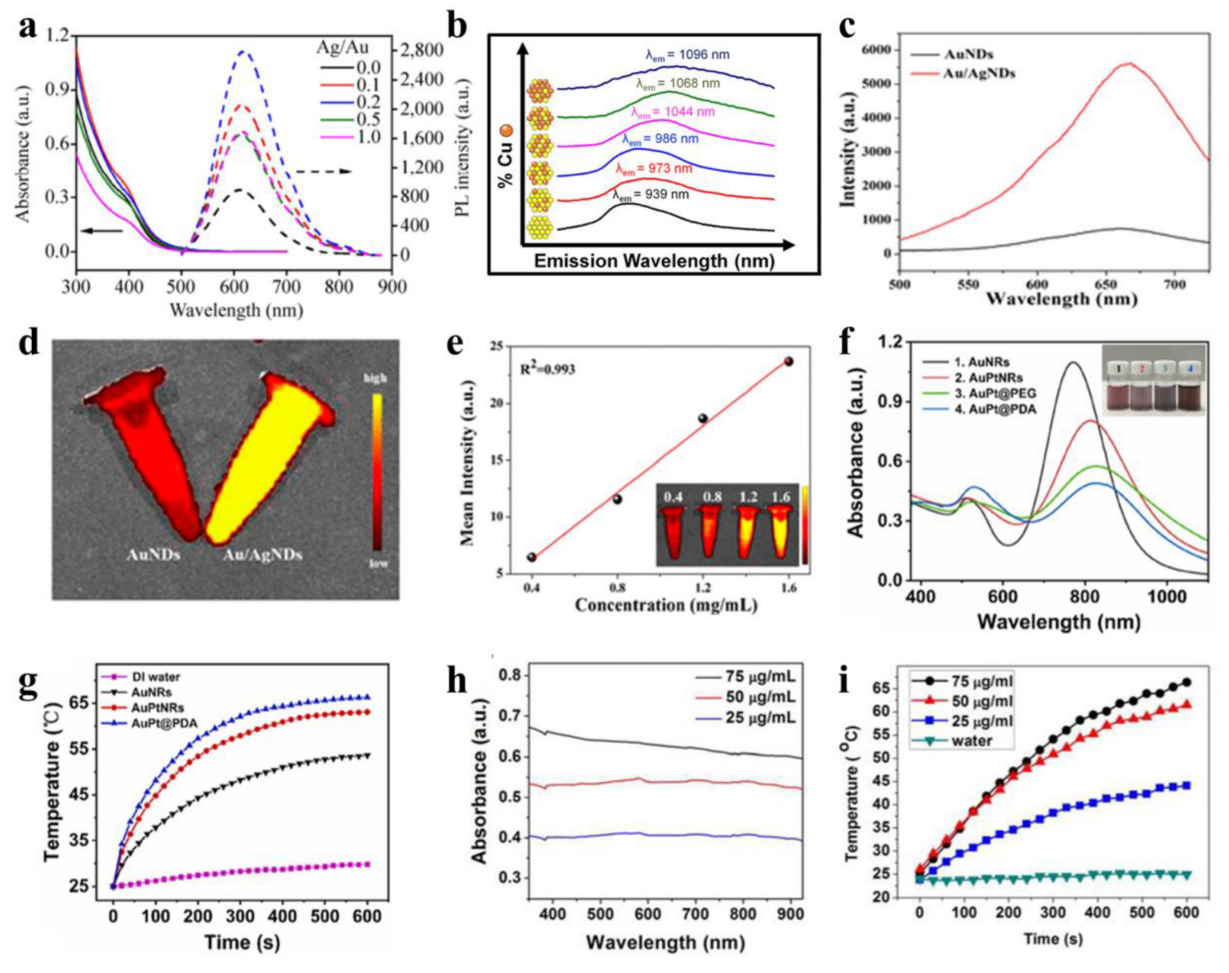
Optical properties of bimetallic nanomaterials. (a) UV-vis absorption and photoluminescence spectra of GS-Au/Ag NCs across varying Ag:Au molar ratios. Adapted with permission from 72. Copyright © 2015, Tsinghua University Press and Springer-Verlag Berlin Heidelberg. (b) Normalized and offset emission spectra of Au_x_Cu_y_NPs, excitation at 360 nm. Adapted with permission from 73. Copyright © 2013 American Chemical Society. (c)-(d) FL spectra and FL imaging photographs of AuNDs and Au/AgNDs. (e) FL intensity trend of Au/AgNDs at different concentrations. Adapted with permission from 7. Copyright © 2023 The Authors. Published by American Chemical Society. (f)-(g) UV-vis absorption spectra and temperature profiles of nanoparticles. Adapted with permission from 74. Copyright 2021 Elsevier B.V. (h)-(i) UV-vis-NIR absorption and photothermal response profiles of PEGylated Au@Pt nanodendrites (NDs) in aqueous solution. Adapted with permission from 75. Copyright © 2017 American Chemical Society.

Bimetallic nanomaterials can greatly promote their fluorescence properties by exploiting following strategies. The addition of a second metal (for example, silver or copper) to the origin metal and exactly adjusting the ratio of bimetallic components can tune their energy band structure, making their emission wavelength switch from the near-infrared I to near-infrared II region, which would extend the application scope of *in vivo* imaging. Additionally, by leveraging the synergistic effect of bimetallic systems (such as the gold/silver system) and ligand engineering (e.g., GSH, mercaptosylated polyethylenimine), the quantum yield and fluorescence intensity can be enhanced. The mechanism lies in the shortening of the excited state lifetime and the inhibition of non-radiative transitions by surface passivation. Yu *et al*. prepared a GSH-coated Au/Ag nanocluster (GS-Au/AgNCs) by microwave irradiation 72. GS-Au/AgNCs exhibit a significantly higher quantum yield (7.8%) than undoped GS-AuNCs (2.2%), along with a markedly enhanced fluorescence emission intensity **(Figure 8a)**. Millstone *et al*. prepared a bimetallic AuCu nanoalloy by introducing Cu into AuNPs. By adjusting the molar ratio of Cu in the nanoalloy, fluorescence emission wavelength of AuCu nanoalloy is redshifted from NIR-I region to NIR-II region **(Figure 8b)**, enabling high-performance NIR nanoprobe functionality for *in vivo* imaging 73. Wang *et al*. prepared multifunctional Au/AgNDs using the ligand mercaptosylated polyethylenimine 7. The fluorescence intensity of Au/AgNDs exhibited a sixfold enhancement compared to AuNDs **(Figure 8c-e)**. This is because after the incorporation of Ag, the excitation state time of the nanodots is shortened, achieving significant amplification of fluorescence emission intensity.

Bimetallic nanomaterials enhance the photothermal performance of single-metal nanomaterials through synergistic modulation of localized surface plasmon resonance (LSPR) effects and hierarchical structural optimization. By precisely designing the compositional ratio, as well as spatial distribution of plasmonic metals and catalytically active metals (such as core-shell or heterostructures), the LSPR absorption peak is redshifted to the near-infrared therapeutic window (e.g., 808 nm), broadening the absorption bandwidth to enhance photon capture efficiency. Furthermore, photoexcited high-energy carriers reduce reaction activation energy, simultaneously amplifying photothermal conversion and catalytic activity to achieve cascaded energy utilization efficiency. For a nanomaterial with no distinct LSPR peaks, the overall absorbance at the target laser wavelength (808 nm) can be directly modulated by compositional engineering, so that effective conversion from photon to thermal energy is promoted. All of these strategies circumvent the major bottlenecks of single-metal nanomaterials such as low near-infrared absorption efficiency, poor thermal conversion efficiency and poor functionalities, offering new channels for precise photothermal therapy.

Bimetallic nanomaterials typically exhibit enhanced photothermal performance due to synergistic amplification of LSPR effects when integrating plasmonic metals (e.g., Au, Ag, Pt, Cu) through crystallization or physical assembly. For PTT, 808 nm NIR laser irradiation is preferentially employed to maximize tissue penetration depth while minimizing damage to healthy tissues 76. In general, the stronger the absorbance of nanomaterials at the laser wavelength, the LSPR can be generated, and the better their photothermal properties. Due to the composition and shape dependent LSPR behavior of bimetallic nanostructures, modulating these parameters provides an effective route to enhance their photothermal effects. Illustrating this concept, Sang and colleagues fabricated dumbbell-shaped Au-Pt bimetallic nanorods (AuPtNRs) via targeted Pt deposition at the ends of Au nanorods 74. As shown in **Figure 8f-g**, the ultraviolet absorption spectrum shows that the longitudinal LSPR peak of AuPtNRs is redshifted, making the absorption peak closer to 808 nm, and the photothermal conversion effect is better, which is conducive to PTT. Following 10-minute laser irradiation, the aqueous dispersion of Au nanorods exhibited a temperature elevation from 27.0 °C to 53.6 °C. In particular, AuPtNRs solution temperature increased to 63.1 ℃, indicating that bimetallic nanomaterials have better photothermal effects. This enhanced performance stems from the close alignment of their LSPR peak with the 808 nm wavelength and broader absorption bandwidth, which increases photon energy capture and enhances thermal dissipation efficiency. For bimetallic nanomaterials lacking distinct LSPR absorption peaks, higher absorbance correlates with increased photon energy absorption and enhanced heat generation under identical irradiation. Pan *et al*. prepared a kind of Au@PtNDs for synergistic treatment of tumor 75. As Pt grows, the absorption of Au@PtNDs moves to the near infrared region, thereby enhancing the effect of PTT. Given the absence of distinct absorption peaks in the spectra, the researchers utilized concentration-dependent absorbance profiles of Au@Pt NDs to validate enhanced LSPR response and photothermal performance **(Figure 8h-i)**.

In the bimetallic system, LSPR synergizes with catalytic properties to mutually enhance both phenomena, resulting in significantly improved photocatalytic efficiency under laser irradiation. Photoexcited plasma nanoparticles produce high-energy carriers on the surface, thereby lowering the reaction activation energy and significantly enhancing catalytic efficiency 71. Bimetallic nanoparticles integrating catalytic transition metals (e.g., Pt, Pd, Cu) with plasmonic noble metals (e.g., Au, Ag) typically exhibit enhanced photocatalytic performance due to synergistic light-harvesting and catalytic activation mechanisms 77, 78. Scaria *et al*. biosynthesized Ag-ZnO nanocomposites employing Quassia indica foliar extracts as a green precursor. The synthesized nanocomposites show that it has excellent photocatalytic activity and biocompatibility, indicating that it has broad application prospects in environmental remediation and cancer therapy 79. Manviri *et al*. synthesized NiO-ZnO, ZnCo_2_O_4_, MnCo_2_O_4_, and CoFe_2_O_4_ nanocomposites using citrus leaf extracts as raw materials by the green route and evaluated their ability to photocatalyze the removal of carcinogenic polycyclic aromatic hydrocarbons 80.

#### 2.3.3. Catalytic properties of bimetallic nanomaterials

Possessing both distinctive physicochemical traits and native enzyme-mimicking functions, nanozymes offer compelling benefits such as excellent stability, simple preparation, low cost, adaptable properties, and ease of storage. Among various nanomaterials, metallic nanomaterials stand out for their excellent catalytic properties, which in medical contexts are described as enzyme-like activity. Extensive research confirms that bimetallic nanomaterials exhibit enhanced catalytic activity compared to monometallic counterparts. Bimetallic nanomaterials enhance catalytic performance through four synergistic mechanisms, overcoming the limitations of single-metal catalysts: (1) Electronic structure optimization through interfacial electron transfer to modulate d-band centers and Fermi levels, lowering activation barriers; (2) Integration of multi-enzyme-mimetic functionalities enabling cascade reactions that overcome monometallic systems' activity constraints; (3) Stimuli-responsive catalysis engineered for tumor microenvironment (pH/H₂O₂) and external trigger (light/temperature) sensitivity, permitting spatiotemporal precision; (4) Nanostructural engineering of core-shell or porous architectures to maximize active site accessibility and enhance catalytic metal redox cycling. At present, the enzyme-like activity of bimetallic nanomaterials has been applied in environmental monitoring, disease diagnosis and treatment.

**Figure 9:**
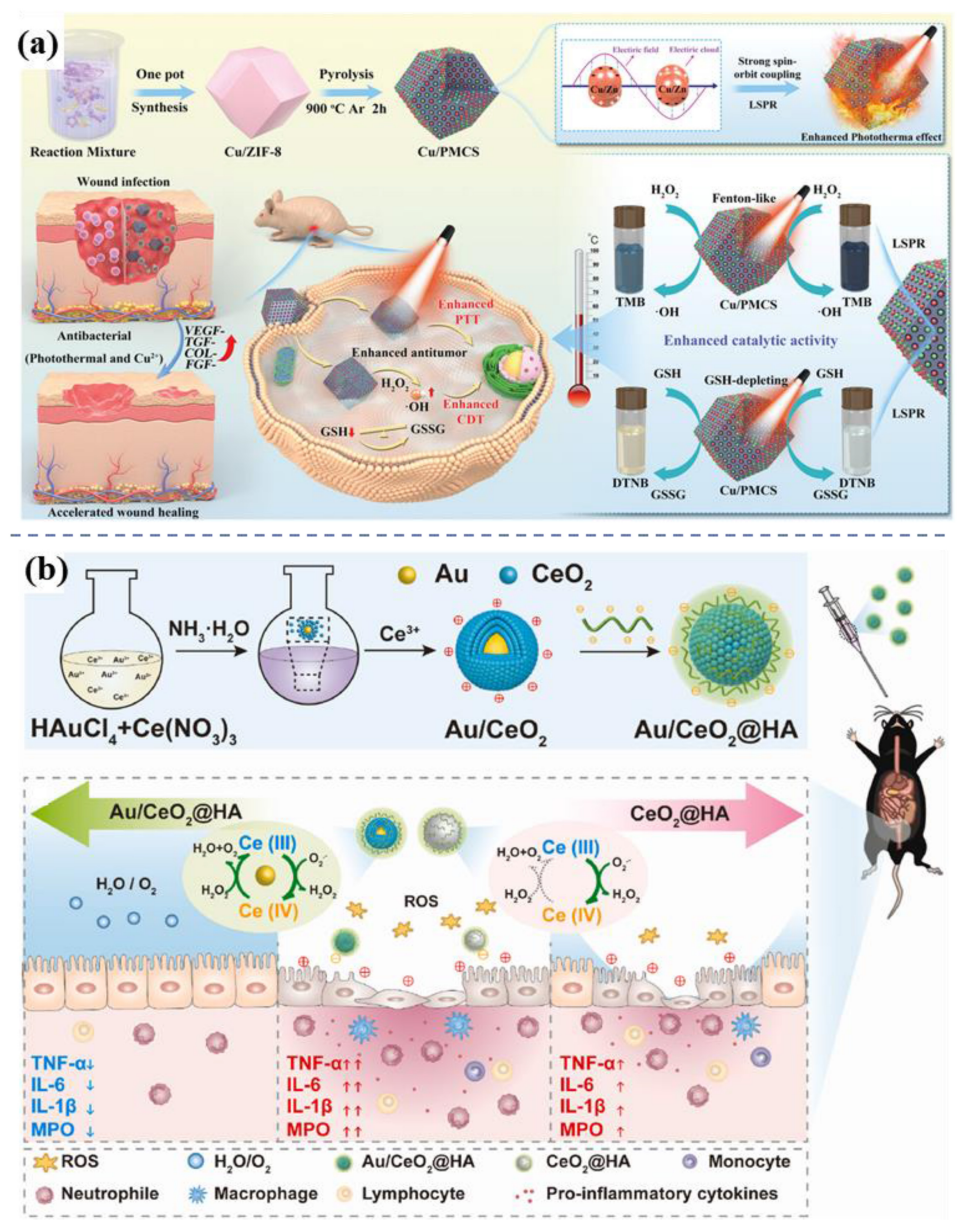
Enzyme-like activity of bimetallic nanomaterials. (a) Schematic illustration of preparing and therapeutic effects of Cu/PMCS. Adapted with permission from 82. Copyright © 2023 The Authors. Advanced Science published by Wiley-VCH GmbH. (b) Schematic illustration of preparing and therapeutic effects of Au/CeO_2_@HA nanozyme for mice with colitis Adapted with permission from 84. Copyright © 2023 The Authors. Publishing services by Elsevier B.V. on behalf of KeAi Communications Co. Ltd.

In cancer therapy, nanozymes are widely applied owing to their diverse enzymatic functions, such as peroxidase (POD), glucose oxidase (GOx), catalase (CAT), and glutathione peroxidase activities. Gold nanomaterials possess a remarkable yet previously underappreciated catalytic potential, manifesting as artificial enzyme activities including those mimicking nucleases, esterases, silicate enzymes, as well as GOx, POD, CAT, and superoxide dismutase. The diverse enzyme-like activities are derived from either the intrinsic properties of the gold nanoparticles or the surrounding functional groups 81. Enhanced photothermal and catalytic activities attributed to LSPR effects are demonstrated by the Cu-Zn bimetallic single-atom material (Cu/PMCS) in Liu's study **(Figure 9a)** 82. The catalytic activity and glutathione depletion capacity of Cu/PMCS in fenton-like reaction are enhanced, and further enhanced with the increase of temperature and LSPR. The vivo/vitro results indicated that Cu/PMCS has potential application prospects in the treatment of melanoma and wound repair. Hu *et al*. prepared a multifunctional RuCuNPs with double enzyme-like activity 83. RuCuNPs leverage the overexpressed H₂O₂ in the tumor microenvironment to both generate O₂, thereby alleviating hypoxia, and produce highly toxic ·OH radicals for tumor cell killing. These actions correspond to their intrinsic CAT-like and POD-like activities, respectively. Li *et al*. reported a nanocomposite consisting of gold nanoparticles dispersed on cerium oxide (Au/CeO₂), which enhanced SOD- and CAT-mimetic activities for the treatment of inflammatory bowel disease **(Figure 9b)** 84. Au/CeO₂@HA, with its core-shell porous structure that enhances antioxidant activity, alleviates colon injury in an acute colitis mouse model by accumulating in inflamed colon tissue and reducing pro-inflammatory cytokines upon oral administration.

Another typical example is Au@PtNPs, where Au@PtNPs shows much higher catalytic activity than single Pt and other bimetallic NPs owing to synergistic and electron transfer effects 85. Density functional theory further confirms a shift in the d-band center of Au following the formation of Au@PtNPs 86. Compared with AuNPs, the surface of Au@PtNPs has a wider d-band and a higher Fermi level, indicating that Au@PtNPs has better catalytic activity. Consequently, Au@PtNPs exhibit superior catalytic activity compared to their monometallic counterparts. Au@PtNPs have peroxidase-like 87, oxidase-like 88 and catalase-like 87 activities, which could catalyze the reduction of H_2_O_2_ and oxygen, as well as catalyze ectopic decomposition of H_2_O_2_ to O_2_.

#### 2.3.4. Stability of bimetallic nanomaterials

The inherent instability of nanomaterials, stemming from their elevated specific surface area and concomitant surface energy, represents a fundamental challenge. Such a constraint can be targeted for bimetallic systems by carefully regulating the metallic composition, atomic configuration and nanoscale structure – reminiscent of the established notion that alloy formation renders stability in normal metallic systems 89. Stability of bimetallic nanoparticles is in full evidence by their outstanding thermal stability, photocorrespondence stability, long-life stability of electrodes, as well as excellent colloidal stability. Naikoo *et al*. summarize the recent advances of bimetallic nanocomposite glucose sensors to be able to achieve rapid and precise glucose detection. They consider the synergistic interaction between different metallic components in bimetallic nanostructures is responsible for more promising measurement reproducibility and excellent practical robustness of these sensors as compared with their single-metallic sensors 90. As stability is a critical factor for medicines, Deng *et al*. synthesised PtRu-PEG BNCs as a stable nanoagent for CT imaging and thermoradiotherap 91.

## 3. Metallic Nanozyme

With the development and rapid expansion of the application of nanoenzyme in many different fields, different kinds of nanozymes are emerging for tumor treatment. Different kinds of nanozymes can be classified into seven categories according to their catalytic mechanism: oxidoreductases, hydrolases, transferases, isomerases, lyases, ligases, and translocases. The curative effect of most nanozymes mainly comes from their oxidoreductase-mimic property. The oxidoreductases include subcategories such as peroxidase (POD), oxidase (OXD), superoxide dismutase (SOD) and catalase (CAT), nanozymes can be classified into several categories according to composition: metal-based, metal oxide-based, metal–organic framework, carbon-based, single-atom and covalent organic framework. This review summarizes the application of metal-based nanozymes in tumor treatment, with the aim of offering research workers complete and systematic information.

Common methods for preparation of metallic nanozymes include chemical reduction, co-precipitation, seed growth, one-pot hydrothermal synthesis, thermal decomposition, and green biosynthesis. Chemical reduction using reducing agents (NaBH_4_ or ascorbate/citrate, etc.) provides excellent flexibility on particle size, morphology and composition, as well as simple and convenient techniques. However, strong reducing agents can generate uncontrolled particle growth, achieving nanostructures with proper nanostructures (such as core–shell) needs mild agents and requiring stepwise addition of these reducing agents. Co-precipitation excels at achieving homogeneous molecular mixing of two or more metal ions at the same time by collective precipitation, is relatively simple and low cost, and can generate nanoparticles with special functional properties (such as magnetic properties). Its downside is sensitivity to the reaction conditions (such as pH, reaction temperature, ionic strength) and potential difficulties in the control of particle size and the avoidance of aggregation without appropriate capping agents. Seed-mediated growth is unparalleled for fabricating more complex and well-defined architectures, such as core-shell, satellite or Janus structures by depositing secondary metals to existing seeds, yielding rapid and very precise control over structure–property relationships. The main disadvantages are the complexity of the process, control over the condition of great precision and the difficulty in making very asymmetric structures, such as Janus NPs, without a specific strategy for seeding symmetry breaking.

Despite worries about side reactions resulting from the incomplete purification, the one-pot hydrothermal method is highly desired due to its simplicity, high yield and cheapness. Thermal decomposition of complex molecules in non-aqueous solvents gives nanoparticles with narrow size distributions and with high compositions fidelity. The thermal method is particularly suitable for noble metal-transition metal combinations unsuitable for being put into water. This outstanding performance, however, often requires inert atmosphere and toxic chemicals, plus self-nucleated by-products needing further purification. Green biosynthesis using biological extracts or biomolecules (for example, DNA) as the reducing or capping agent works under mild and environmental conditions, follows the principle of sustainability and enables large-scale production. Its prominent drawbacks are the challenge of controlling uniformity of particles (size/morphology), dependence on possibly unreliable biological materials, limiting scalability, and inadequately checked biosafety profiles needing future consideration. Seed-growth guarantees structure-control, at the cost of complexity. Hydrothermal/thermal methods can give high yields/distributions, but the reaction control/purity problems are common and biosynthesis favours green chemistry but ineffectual in terms of uniformity and scalability. Progress in this field will probably lie in hybrid routes and computational design for accuracy improvement.

Compared with natural enzymes, metallic nanozymes have superior performance in terms of activity and catalytic efficiency. Fan *et al*. adopted a surface engineering strategy to modify Ru nanozyme with polystyrene sulfonate (PSS), achieving a peroxidase-like specific activity as high as 2820 U mg^-1^, which is twice that of natural horseradish peroxidase (HRP, 1305 U mg^-1^). Mechanism studies have shown that PSS accepts a negative charge from Ru, reducing the affinity of Ru for ·OH, thereby enhancing the catalytic activity. This nanozyme has been successfully applied to the immune detection of human alpha-fetoprotein, and its detection sensitivity has been increased by 140 times compared with the traditional ELISA based on HRP, directly proving that metallic nanozyme can surpass the performance of natural enzymes in practical applications 92. Ji *et al*. reported a FeN_3_P single-atom nanozyme (SAzyme), which achieved catalytic activity and kinetic properties surpassing those of natural horseradish peroxidase by precisely regulating the electronic structure of the active center. Experimental data show that the catalytic efficiency of FeN_3_P-SAzyme is 1.40 × 10^8^ M^-1^ min^-1^, and the Km value is 2.06 × 10^-3^ mM. However, the catalytic efficiency of natural HRP was 1.15 × 10^7^ M^-1^ min^-1^, and the Km value was 5.55 mM. Furthermore, the nanozyme was consistent with Michaelis-Menten kinetics, and the source of the high enzyme-like activity was revealed through density functional theory calculations 93. Monometallic (Au, Cu, Pt), bimetallic nanozymes as well as cerium oxide variants and so on have potent enzyme-mimicking catalytic activities for multimodal anti-cancer application, especially metabolic intervention and CDT 94.

**Figure 10:**
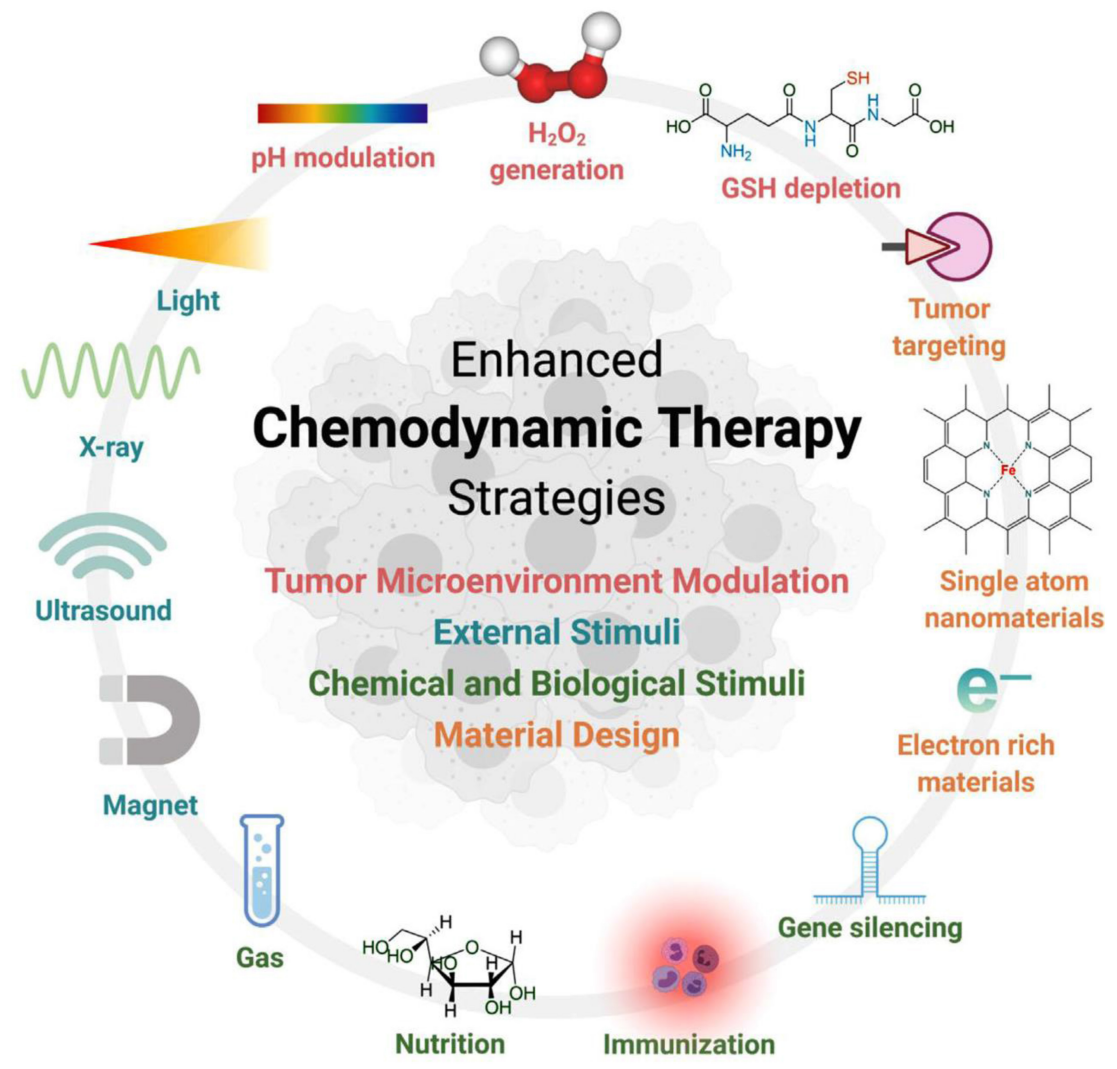
Overview of the strategies to improve CDT. Adapted with permission from 97. Copyright © 2022 The Authors. Exploration published by Henan University and John Wiley & Sons Australia, Ltd.

CDT represents an emerging therapeutic paradigm that leverages Metallic nanozymes-mediated Fenton/Fenton-like catalysis to convert tumoral H_2_O_2_ into cytotoxic reactive oxygen species (ROS). This process induces apoptotic cascades via proteomic denaturation, membrane lipid peroxidation, and genomic instability 95, 96. CDT relies on overexpression of H_2_O_2_ and mild acidity of TME, and is highly specific to cancer tissue, while having little or no toxicity to normal cells. As the study progressed, the researchers developed a number of strategies to optimize Fenton reaction and CDT performance. These strategies focus on the regulation of TME (reducing pH, increasing H_2_O_2_ concentration, and depleting GSH, etc.), the use of external stimuli (light, ultrasound, etc.), chemical and biological stimuli (gas molecules, immune adjuvants, gene silencing, and nutrient composition), and optimization of nanoparticle design **(Figure 10)** 97.

**Figure 11:**
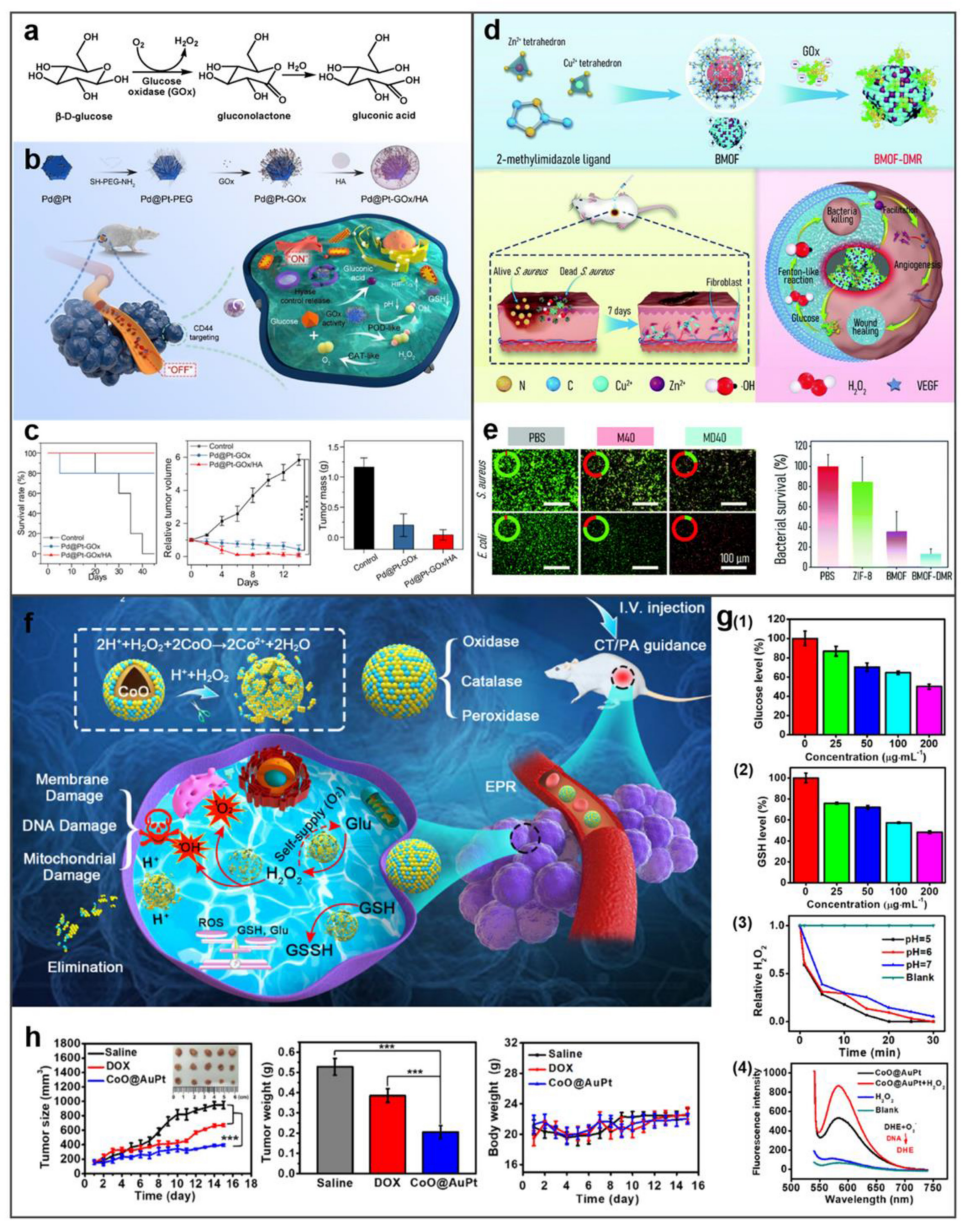
Enzyme-mediated tumor therapy. (a) Molecular mechanism of GOx-catalyzed dual-substrate depletion. (b) Tumor-targeted cascade catalysis by Pd@Pt-GOx/HA nanozymes enabling starvation-amplified CDT. (c) Nanozyme-enhanced starvation therapy inhibits 4T1 tumor growth. (d) BMOF-DMR promotes bacteria-infected wound healing and eliminates bacteria. (e) BMOF-DMR eliminates bacteria in infected wounds. (f) Schematic depiction of CoO@AuPt NPs enabling enhanced CDT. (g) The enzyme-like activity of CoO@AuPt. (h) Therapeutic effect of CoO@AuPt. Adapted with permission from 58. Copyright © 2022 by the authors. Licensee MDPI, Basel, Switzerland.

As a metabolic-targeting mode, starvation therapy exploits GOx-mediated glucose depletion-induced death of the TME metabolites for decreasing ATP synthesis in tumors. The GOx-mediated substrate depletion is an enzyme-mediated limitation method, exploiting the dramatically elevated glycolytic capacity (Warburg effect) of cancer cells for starvation therapy specificity 98. The GOx-mediated catalytic cycle oxidizes glucose while consuming dissolved oxygen, generating gluconate and H_2_O_2_. The two depletion processes inhibit both glycolysis and oxidative phosphorylation, enforcing TME selective bioenergetics death** (Figure 11a)**. The generated H_2_O_2_ amplifies tumoral oxidative stress while accumulating hydroxyl radicals (·OH) from peroxidase activity of metallic nanozymes for targeted cell killing. Concurrently, gluconic acid production acidifies TME, triggering pH-responsive therapeutic modalities that potentiate anti-tumor efficacy 99. Professor Wang Weilin's team combined AuNPs with GOX-like effect *in situ* on a metal-organic framework (MOF) for cascaded CDT and starvation therapy, solving the problem of poor CDT effect alone and enhancing the therapeutic effect of liver cancer.

For instance, Zheng *et al*. engineered a stimulus-responsive nanoreactor (Pd@Pt-GOx/HA) with switchable enzymatic cascades, leveraging platinum-palladium heterojunctions for dynamically regulated catalysis 100. As shown in **Figure 11b**, CD44-overexpressing tumors undergo selective HA-mediated endocytosis of Pd@Pt-GOx/HA, triggering lysosome-activated nanoreactor disassembly and subsequent Pd@Pt-GOx release. Subsequently, GOx consumes glucose and oxygen for starvation therapy. This process generates H_2_O_2_ and creates an acidic microenvironment, which activates the Pd@Pt nanozyme. The activated nanozyme then catalyzes H_2_O_2_ decomposition into ⋅OH to realize CDT. In tumor-bearing mouse models, Pd@Pt-GOx/HA treatment resulted in remarkable tumor eradication and survival time extension **(Figure 11c)**. Extending beyond oncological applications, this strategy demonstrates significant antimicrobial potential. Peng *et al*. developed a Zn/Co bimetallic MOF microreactor (BMOF-DMR) capable of disrupting bacterial membranes through catalytic cascade reactions **(Figure 11d and 11e)** 101. Fu *et al*. developed biomimetic CoO@AuPt nanozyme that combines cascade reactions around the tumor microenvironment to efficiently produce the ROS, and realize the stimuli-free initiation of CDT **(Figure 11f)** 102. As shown in **Figure 11g**, CoO@AuPt has multiple catalytic properties: glucose or glutathione depletion, catalase mimetic and ROS generation. Animal studies validated its tumor-specific ablation efficacy without harming adjacent tissues, demonstrating high therapeutic selectivity **(Figure 11h)**. Altogether, all these studies show the powerful CDT ability of metal-based nanozymes as the catalyst for cancer treatment. This multienzyme-featured strategy for cancer treatment has reached an emerging field of targeted oncology therapy.

Additionally, elevated temperature could enhance the kinetics of Fenton reaction, so photothermal effects often work synergistically with Fenton reaction to enhance CDT. Huang *et al*. developed a nanocomposite consisting of AuNRs, SiO_2_ and MnO_2_ (GNR@SiO_2_@MnO_2_, GSM) 103. Within the endogenous acidic tumor microenvironment, the MnO_2_ layer can be degraded into Mn^2+^, and the released Mn^2+^ participates in the Fenton-like reaction to achieve CDT. PTT mediated by near infrared laser was used to kill cancer cells *in vitro*, and photothermal enhanced the effect of Fenton-like reaction. Combined therapy effectively inhibited tumor growth. In another study, Yin *et al*. developed a hollow Mn/Cu/ZnMOF loaded with ICG and MnO_2_ coatings for image-guided PTT, PDT, and CDT multimodal therapy. The laser-mediated photothermal effect can generated localized hyperthermia and accelerate the formation of ⋅OH to enhance CDT 97. Wang *et al*. developed Au/CuNDs that integrate potent nanozyme and photothermal activities into one platform to enable PTT, photothermal-enhanced CDT, and synergistic chemotherapy for cancer therapy 104.

## 4. Metallic Nanomotor

The abnormal vascular structure, high interstitial pressure and dense extracellular matrix network in the tumor tissue inhibit the intratumoral penetration and intracellular internalization of nanomaterials in the TME 105, 106. In addition, lysosomal escape has also become a bottleneck for nanomaterials, as the acidic environment inside the lysosome poses a risk of nanomaterials degradation 107. Therefore, enhancing the penetration of nanomaterials and lysosomal escape is of crucial importance. Metal-based nanomotors with autonomous movement capabilities can overcome these shortcomings and increase tumor penetration at the nano scale at the same time. Featuring an asymmetric structure with distinct physicochemical properties on each side, nanomotors can convert chemical or external energy into mechanical motion, functioning as self-propelled nanodevices 108, 109. Leveraging their versatile biologically functional surfaces, nanomotors hold great potential to enhance tissue penetration and boost therapeutic outcomes in complex disease treatment 10.

**Figure 12:**
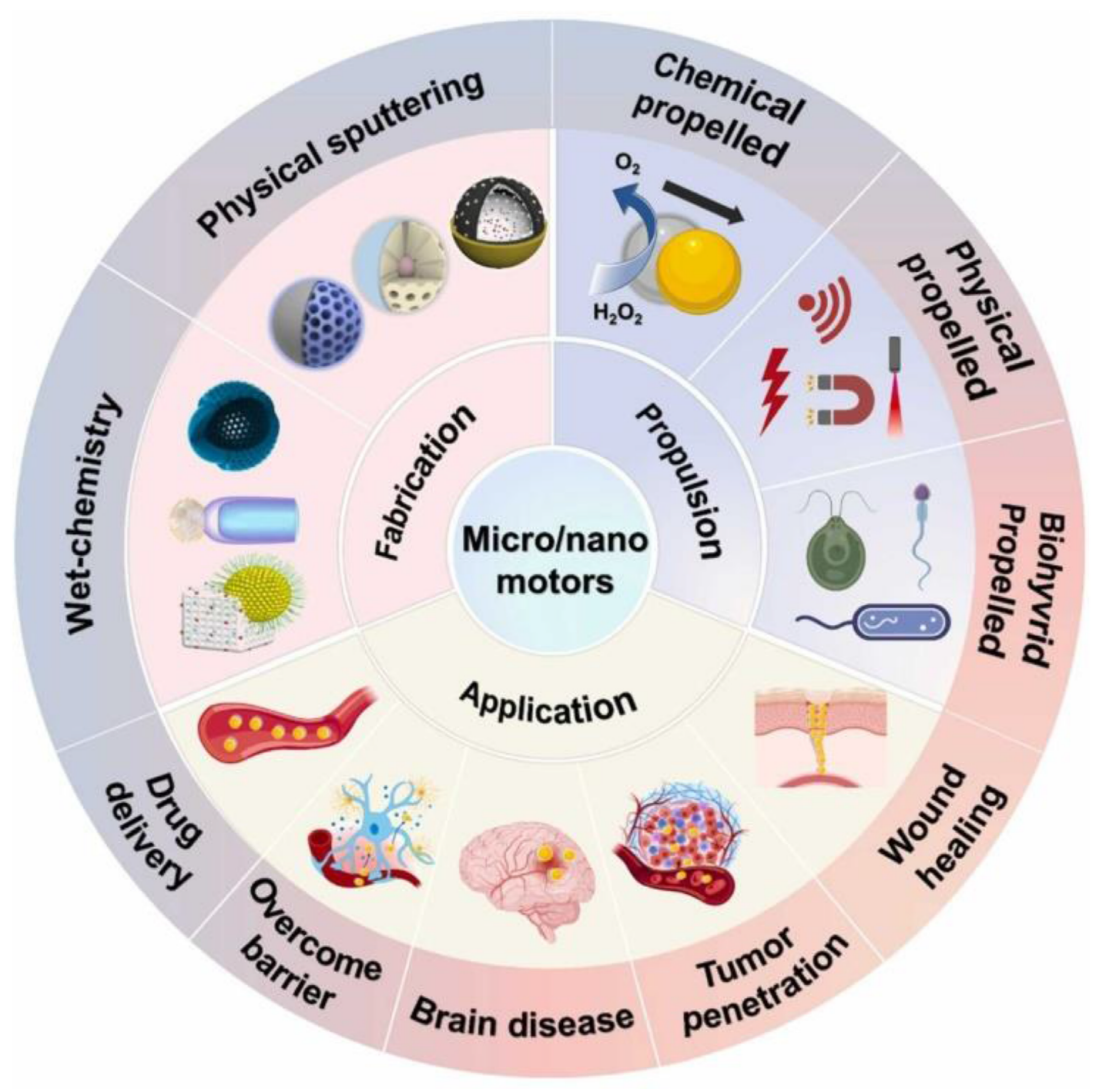
Schematic illustration of fabrication strategy, propulsion mechanism, and biomedical application of nanomotors. Adapted with permission from 110. Copyright © 2024 Elsevier Ltd.

Manufacturing metallic nanomotors depend critically on the creation of asymmetric Janus structures to drive the direction of propulsion. Physical deposition (primarily sputtering) is the most commonly method. It is to vaporize the target metal (e.g., Pt, Au, alloys) and deposit it onto one side of substrate nanoparticles (e.g., silica, carbon spheres) to pattern precision asymmetric coatings. This is the best method for precise structural control, giving well defined Janus structures required for propulsion. However, physical deposition has severe disadvantages—including the large dependence on the expensive and complicated equipment (sputtering equipments), limiting it to a small-scale manufacturing process. Wet-chemical synthesis methods are a desirable choice for large scale, cheap preparation of metallic nanomotors, making them more viable for biomedical applications. These methods obtain the asymmetry by controlling the chemical processes, such as the spatial ligand competition or specific assembly procedures. This method could generate asymmetric structures such as eccentric hollow tadpoles, AuNR-TiO_2_ rods, parachute-like Pt-poly(divinylbenzene) particles. Although wet chemistry can achieve high yields and controllable properties, this method also has drawbacks: wet chemistry requires strict control over multi-step reaction pathways and complex post-treatment, which increases the complexity and time of the process. Achieving uniform morphology and size distribution remains difficult due to the inherent complexity of MNM structures. Recently, Ye *et al*. provided a comprehensive overview of nanomotor fabrication, propulsion mechanisms, and biomedical applications **(Figure 12)** 110.

**Figure 13:**
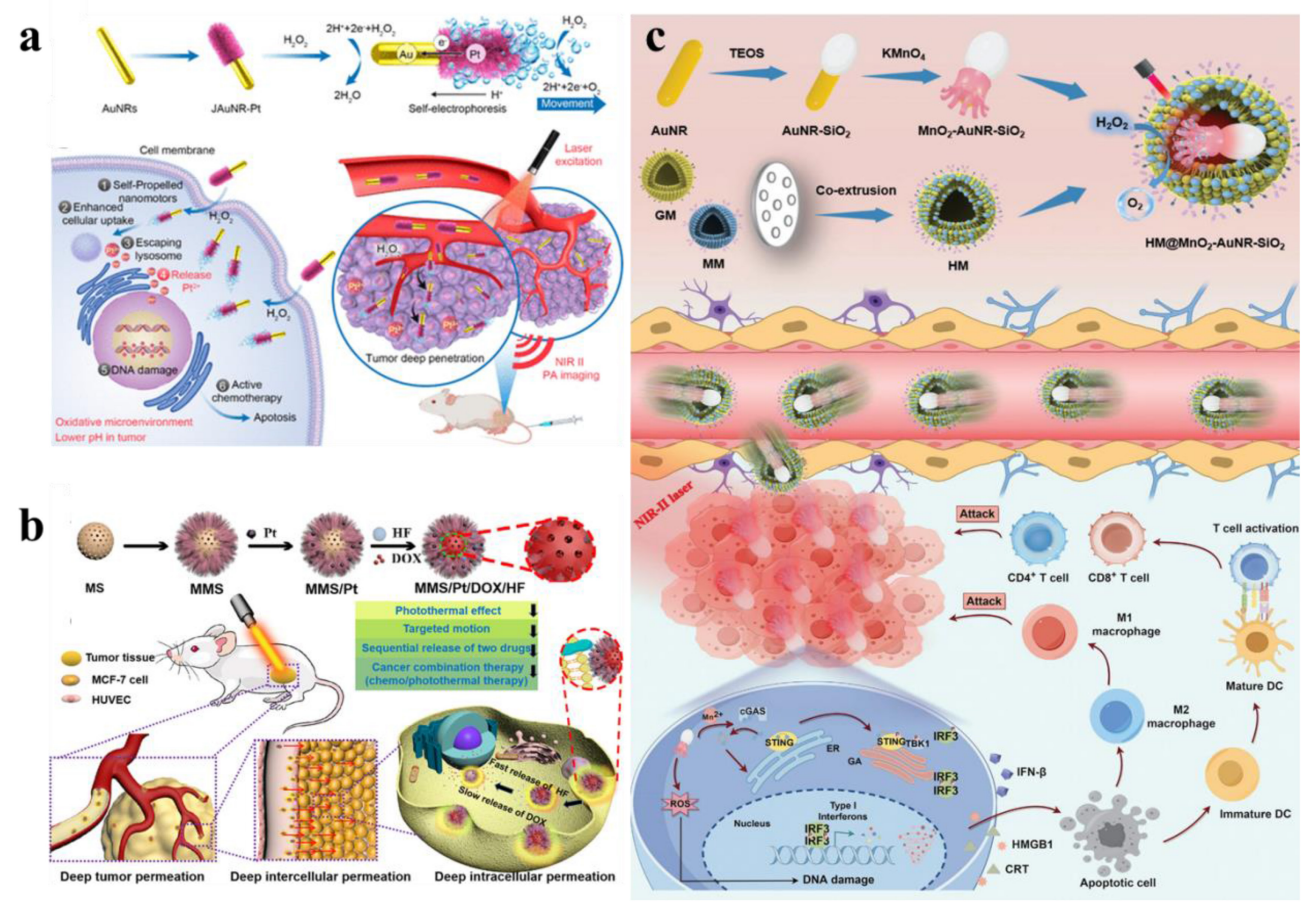
Metal-based nanomotor-mediated tumor therapy. (a) JAuNR-Pt nanomotors for NIR-II PA imaging and antitumor therapy. Adapted with permission from 113. Copyright © 2022 American Chemical Society. (b) MMS/Pt/DOX/HF nanomotors for cancer therapy. Adapted with permission from 115. Copyright © 2020 Wiley-VCH Verlag GmbH & Co. KGaA, Weinheim. (c) Schematic illustration of HM@MnO_2-_AuNR-SiO_2_ nanomotor fabrication and its application in targeted catalytic immunotherapy for GBM. Adapted with permission from 116. Copyright © 2024 Wiley-VCH GmbH.

Leveraging the outstanding enzyme-like activity and responsiveness to external stimuli such as light, electricity, ultrasound, and magnetism of metallic nanomaterials, metal-based nanomotors are typically categorized into two types: those driven by artificially controlled external fields and those powered by chemical fuels present in the system, including water, hydrogen peroxide, urea, and hydrogen 111. Enzyme-powered nanomotors, one of the most prevalent chemically fueled motors, harness the efficient biocatalysis of endogenous biofuels within biological hosts. This process generates a propulsive force strong enough to overcome random Brownian motion, enabling autonomous movement 112. For instance, Li *et al*. fabricated a H_2_O_2_-driven Janus gold nanorod-platinum (JAuNR-Pt) nanomotor. This nanoscale motor not only enhances NIR-II photoacoustic imaging of deep tumor tissues but also enables effective tumor therapy **(Figure 13a)** 113**.** Tang *et al*. developed light-propelled nanomotors incorporating polyoxometalate nanozymes, enabling targeted and synergistic photothermal-catalytic cancer therapy 114. Conjugated polydopamine confers light-driven self-propulsion to nanomotors. When combined with NIR irradiation and epidermal growth factor receptor antibody assistance, these nanomotors achieve targeted tumor accumulation and penetration, realizing efficient synergistic photothermal catalytic therapy. This strategy overcomes the physiological instability of enzyme-driven nanomotors and enables motion-enhanced antitumor efficacy. Wan *et al*. designed and fabricated a multidrug near-infrared light-driven nanomotor with autonomous movement, targeting ability, stacked porous structure, and suitable for cancer chemotherapy/photothermal therapy **(Figure 13b)** 115. The results of tumor elimination *in vivo* verified that the movement behavior of nanomotors can be greatly promoted through various treatment methods, thereby significantly facilitating tumor elimination. Hu *et al*. developed NIR-driven nanomotor ZnO2@PDA-Fe (Z@P-F) to enhance tumor penetration and treatment 9. In their research, they compared them with traditional nanoparticles. Z@P-F nano-motors combined with near-infrared light achieve the best tumor penetration depth. This is because the movement induced by near-infrared activation and the destruction of the extracellular matrix structure of tumor cells through thermal effects enhance the diffusion of nanomotors in the extracellular matrix, thereby promoting the deep penetration of tumors.

As research advances, nanomotors with dual or multiple driving forces have been developed. Ye *et al*. engineered a dual-driven heterojunction nanomotor (HM@MnO_2-_AuNR-SiO_2_) cloaked with biomimetic hybrid cell membranes for targeted glioblastoma therapy, which bypasses the blood-brain barrier by mimicking GBM and macrophage membrane properties and achieves deep tumor treatment via NIR-II light and O₂ bubble dual-driven propulsion **(Figure 13c)** 116.

## 5. Metallic Nanomaterials in Cancer Diagnosis: Advances and Applications

Advancements in imaging technology are pivotal for disease diagnosis. Benefiting from excellent physicochemical properties, biocompatibility, and pharmacokinetic behavior, AuNPs are outstanding nanoprobes for such applications 12. Ultrasmall AuNPs with quantum confinement-induced energy level quantization of excited states give size-tunable fluorescence extending to the NIR-II region (1000 ~ 1700 nm). Together with the intrinsic fluorescence, ultrasmall AuNPs with kidneys as being the natural clearance pathway, little nonspecific uptake and EPR effect are new types of optical probes with potential to address critical challenges in clinical FL imaging. Due to the single electron transfer, the ultra-small AuNPs show strong absorption bands in the visible to NIR part of the electromagnetic spectrum, conferring powerful photoacoustic imaging (PAI) capabilities 117. As a high-Z material, AuNPs have high X-ray absorption and function as a contrast medium for CT imaging 118. In addition, the surface of AuNPs can be engineered through functionalization, conjugation, or alloy formation with other contrast agents (e.g. Gd^3+^, ^198^Au, and ^64^Cu) to enable multimodal imaging capabilities.

### 5.1. FL imaging

FL is a very important technique for disease diagnosis and therapy, and AuNPs with good photoluminescent properties have extensive applications in FL imaging. Among various probe designs, FL probes based on Au–S bonds are the most widely used designs owing to their simple fabrication and operation processes 119. Various surface-modified AuNPs have been used in bioimaging, among which the most common ones are those bound to peptides or proteins, as they possess excellent birecognition ability, high biocompatibility and adjustable targeting ability 120. Wu *et al*. prepared a bovine serum albumin-gold nanocluster (BSA-AuNCs) as a near infrared imaging agent, exhibiting strong potential for tumor imaging 121. It was further confirmed that BSA-AuNCs enriched in tumour regions owing to the EPR effect through both *in vitro* and *in vivo* studies. Wang *et al*. used GSH-capped silver nanoclusters as templates and prepared AuNCs by electrochemical reduction to improve their fluorescence quantum yields 122. The engineered GSH-AuNCs demonstrate utility for label-free FL imaging of malignant cells *in vitro*, serving as biocompatible nanoprobes for visualizing subcellular structures. In addition, the potential bioimaging applications of fluorescence AuNPs can be further expanded by synthesizing them *in situ* in organisms 123. In addition to single AuNPs, AuNPs-based composites are also being developed for biological imaging. For example, cationic polymers polyacrylamine hydrochloride and GSH-AuNCs significantly enhance fluorescence through aggregation induced luminescence (AIE) effect after self-assembly 124. *In vitro* results showed that the cellular uptake capacity of self-assembled nanocomposites is greatly enhanced compared to GSH-AuNCs alone.

To achieve targeted cell imaging, recognition sequences such as folate (FA), aptamers, targeted peptides, and antibodies were modified on the surface of AuNPs by surface functionalization 125, 126. FA-modified GSH-AuNCs exhibited enhanced tumor-targeting efficacy in FL imaging, leveraging the overexpression of folate receptors on cancer cells 127. Complementarily, Vankayala *et al*. engineered nucleus-localizing TAT peptide-AuNC conjugates through amide-bond linkage between AuNCs and HIV-1 transactivating transcriptor (TAT) peptide, enabling real-time tracking of intranuclear dynamics in malignant tissues 128. In addition, Chen *et al*. conjugated AuNCs to α_v_β_3_ integrin-specific cyclic arginine-glycine-aspartic (RGD) overexpressed on the surface of tumor cells as well as high-affinity AS1411 aptamers overexpressed in the cytoplasm and nucleus of tumor cells 129. Wang *et al*. developed an LHRHa-conjugated AuNCs nanoassembly through covalent coupling of polyethyleneimine functionalized gold nanoclusters with luteinizing hormone-releasing hormone analogues (LHRHa), establishing a receptor-targeted theranostic platform 130. Thanks to the excellent FL and CT imaging functions of gold, as well as the targeting specificity of LHRHa, Au-LHRHa can achieve specific detection of prostate cancer. Functionalization with fluorescent dyes endowed the nanoplatform with strong tumor-targeting ability, demonstrating its promise for clinical tumor imaging applications.

**Figure 14:**
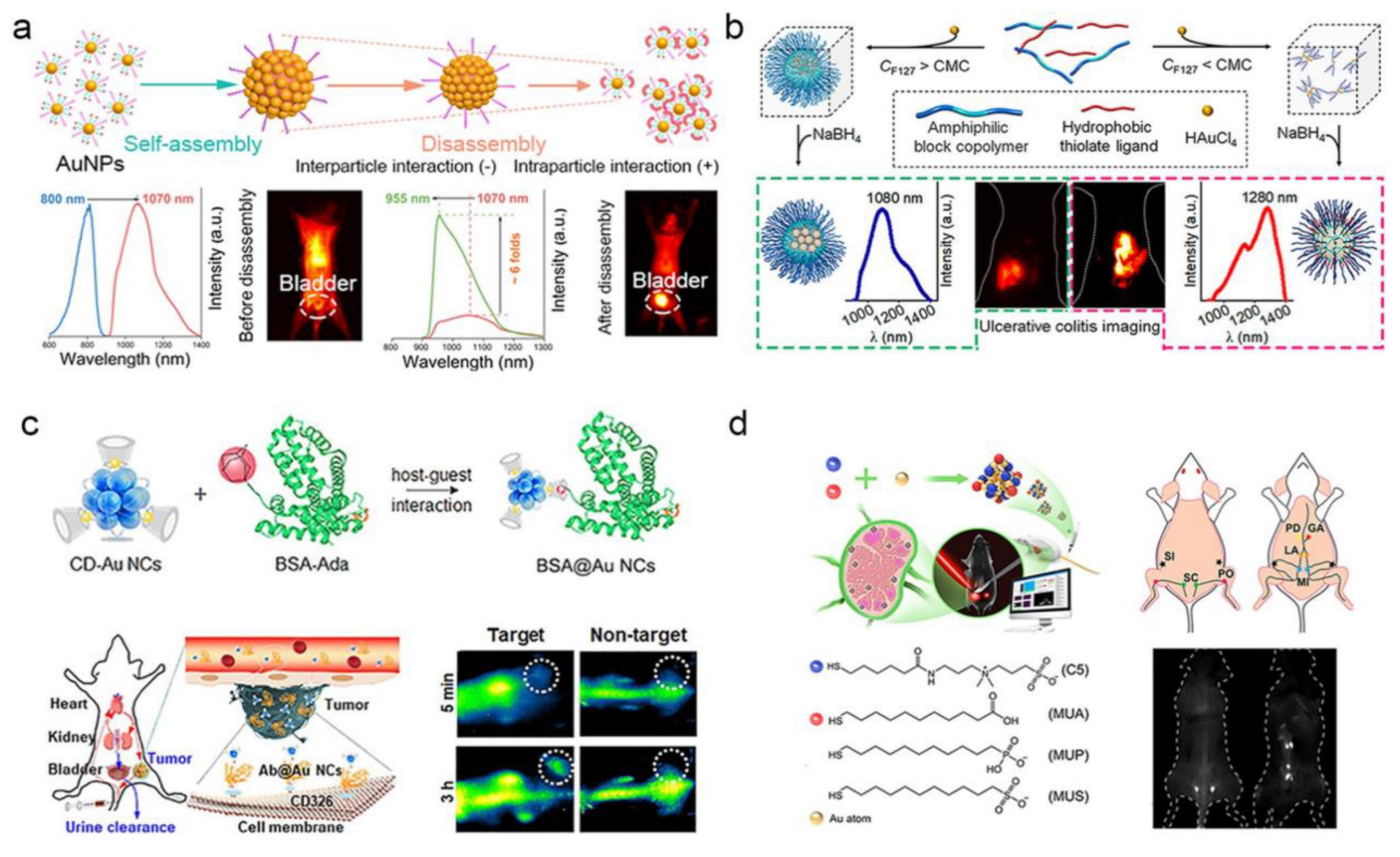
NIR–II fluorescence imaging of AuNPs. (a) Stimuli-Responsive AuNPs with Disassembly-Induced Emission for NIR-II Imaging. (b) Environment-responsive luminescent AuNPs engineered via hydrophobic interaction modulation for real-time colitis imaging in the NIR–II window. (c) Precision tumor-targeted NIR–II imaging using Avidin-Biotin complex-functionalized gold nanoclusters as protein-specific biolabel. (d) Theranostic AuNCs for lymphatic metastasis: integrated receptor targeting, NIR–II image-guided resection, and adjuvant chemodrug delivery. Adapted with permission from 4. Copyright © 2023, The Author(s).

Advancements in NIR–II FL imaging have demonstrated transformative potential for biomedical applications, effectively mitigating fundamental limitations-including strong tissue absorption, autofluorescence interference, and photon scattering-that constrain conventional optical techniques. This modality enables deep-tissue penetration (> 3 mm) thereby establishing a paradigm shift in high-contrast *in vivo* visualization 131. Using a simple coating strategy with organic silane and hydrophilic thiolated PEG, Liu *et al*. prepared NIR-II AuNP nanoassemblies that show remarkable emission enhancement upon disassembly 132. This provides a convenient way to design high-emission AuNPs nanoassemblies for bioimaging **(Figure 14a)**. In addition, Liu *et al*. using amphiphilic block copolymers with controllable hydrophobic interactions as templates, redshifted the emission wavelength of AuNPs and enhanced their biological interactions through a simple strategy, and enhanced affinity for damaged intestinal mucosa in colitis imaging 133 **(Figure 14b)**. These findings establish a paradigm for engineering luminescent AuNPs with programmable NIR-I/II redshift characteristics, enabling enhanced biological interactions through precisely modulated nano-bio interfaces.

Recent advances have enabled the rational design and controlled synthesis of diverse ultra-small AuNPs exhibiting intrinsic NIR-II fluorescence, with systematic evaluation of their imaging performance metrics. Cheng *et al*. synthesized Au_25_(SG)_18_ nanoclusters with an emission peak at 1050 nm. These nanoclusters exhibit high-affinity binding to hydroxyapatite via carboxyl-calcium coordination for active accumulation in osteotropic bone tissues, achieving a signal-to-background ratio of up to 4.35 at 24 h *in vivo* fluorescence imaging to distinguish the spine from surrounding soft tissues 134. As shown in **Figure 14c**, Yang *et al*. developed cyclodextrin-stabilized AuNCs (1.85 nm) emitting at 1050 nm in the NIR-II region for protein and antibody labeling 135. Jiang *et al*. engineered a library of surface-tailored gold nanoclusters AuNCs (1.2 nm), exhibiting NIR-II fluorescence emission (1000-1100 nm) through multiligand capping strategies 136. These AuNCs achieved high-contrast FL imaging of lymph node metastases, demonstrating sustained signal retention at lesion sites (> 3 h) with a signal-to-background ratio (SBR) of 60 **(Figure 14d)**. Dai *et al*. synthesized a GSH-AuNCs (1.6 nm) exhibiting an emission maximum at 1090 nm within the NIR-II biological window 137. After being functionalized by phosphorylcholine, making it a "super-stealth" probe that does not bind to serum proteins like indocyanine green (ICG) and does not have non-specific bone accumulation like GSH-AuNCs.

### 5.2. CT imaging

CT remains a predominantly employed noninvasive diagnostic modality in contemporary clinical practice, offering high-resolution cross-sectional visualization across diverse medical disciplines 138. However, clinically commonly used contrast agents, such as iodine small-molecular agents have such problems as short blood circulation time, limited modifiability as well as toxic side effects 139, 140. As high Z materials (Au [Z = 79], iodine [Z = 53]), AuNPs exhibit superior X-ray attenuation relative to iodine-based agents, with mass attenuation coefficients of 5.16 cm^2^/g (Au) versus 1.94 cm^2^/g (I) at 100 keV. 141, 142, so AuNPs are suitable to function as an excellent CT contrast agent. Meanwhile, AuNPs have an ultra-small size (< 5 nm), longer cycle time, and better biocompatibility, making them excellent candidate for CT imaging contrast agents. Blum *et al*. developed a novel probe targeting cathepsin for CT imaging of tumor 143. The results indicated that the size of gold nanoparticles and the number of targeted fragments were negatively correlated with CT signals at tumor sites. Protein-modified gold nanoclusters were also studied as CT contrast agents. Zheng *et al*. engineered GSH-AuNCs as CT contrast agents, enabling real-time monitoring of renal clearance kinetics via noninvasive imaging within 24 h post-intravenous administration in murine models. The results confirm efficient systemic elimination of GSH-protected AuNCs, with liver accumulation as low as 3.7% and urinary recovery exceeding 50%, indicating predominant renal clearance 144. Wang *et al*. prepared AuNDs with outstanding CT/FL dual-mode imaging performance, facilitating rapid and accurate detection of spinal cord injury sites 145. Due to their ultrasmall dimensions and weak interaction with serum proteins, the nanoclusters show negligible hepatic and splenic uptake, leading to improved CT imaging contrast.

**Figure 15:**
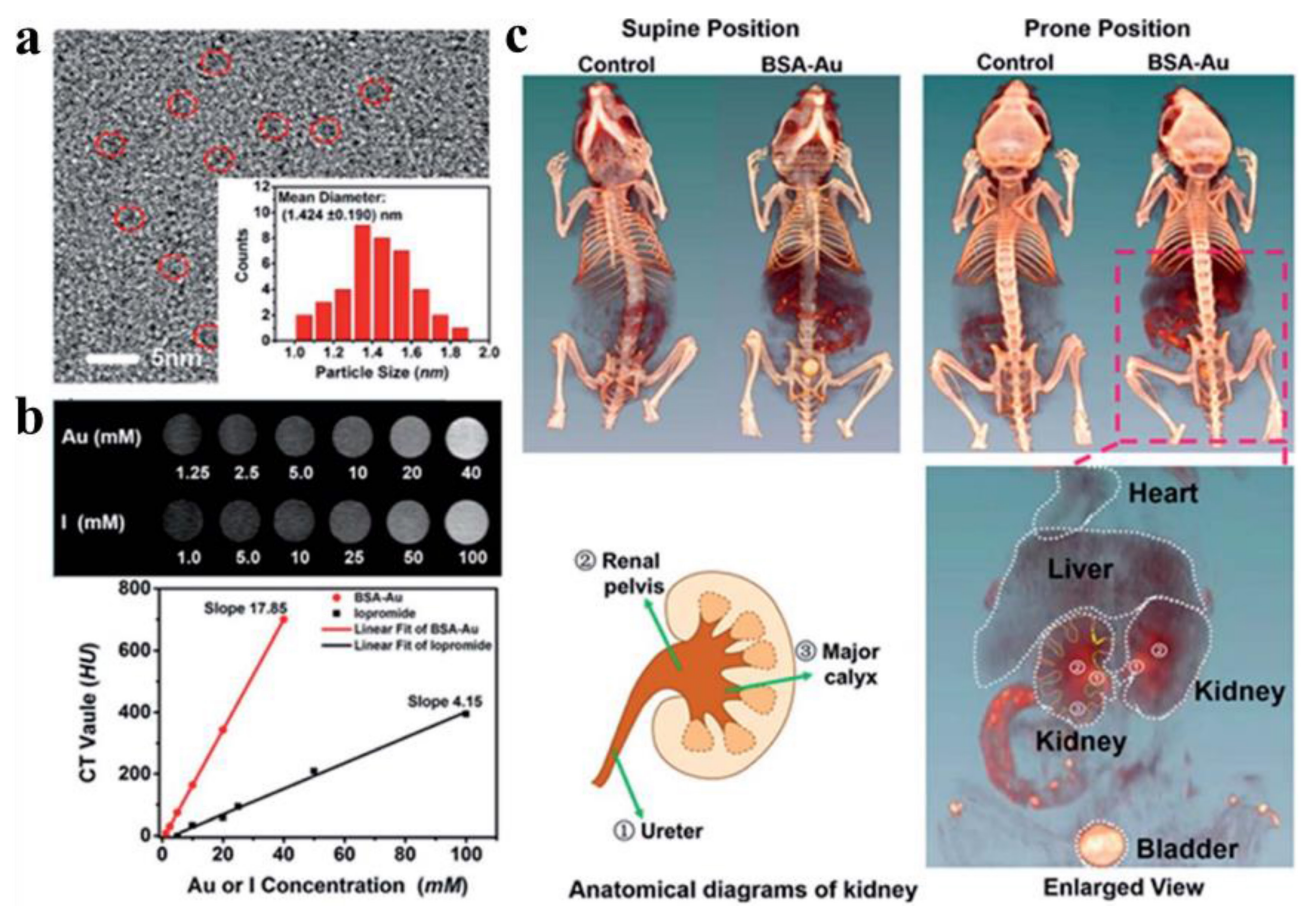
CT imaging of nano-Au. (a) HRTEM image of BSA–Au. (b) Comparison of CT images and HU values between BSA–Au clusters and iopromide solution *in vitro*. (c) At 2 h post-injection, CT images of mice with and without BSA–Au cluster administration *in vivo*. Adapted with permission from 146. Copyright © 2015, American Chemical Society.

Gao *et al*. developed a BSA-protected AuNCs for CT imaging of the kidney 146. Following optimization of the BSA-to-gold ratio, the synthesized AuNCs exhibit enhanced fluorescence properties and improved X-ray attenuation capabilities. *In vitro* CT imaging revealed that the HU value slope of the synthesized AuNCs at equivalent concentrations was 4.3 times higher than that of the clinical CT contrast agent iopromide **(Figure 15).** *In vivo* studies demonstrated that AuNCs provide effective CT contrast enhancement, distributing predominantly in the liver, spleen, and kidneys, with primary renal clearance. The agent delineated murine renal anatomical structures via CT imaging, clearly visualizing the kidneys and ureters. Chen *et al*. reported iodinated gold nanoclusters (AuNCs@BSA-I) with FL and CT dual-mode imaging function synthesized using BSA and chloramine-T. Basilion *et al*. synthesized ultra-small AuNPs functionalized with prostate-specific membrane antigen for targeted CT imaging and RT enhancement of prostate cancer 147. The demonstrated properties of AuNPs and collective findings suggest their potential as future CT contrast agents.

### 5.3. MR imaging

As a non-invasive clinical imaging method, MRI has been used in the analysis and imaging of various brain, vascular, musculoskeletal diseases and tumors 148, 149. Researchers have developed a number of nanomaterials as MRI contrast agents, mainly superparamagnetic magnetite and paramagnetic complexes of Gd^3+^ or Mn^2+^. They provide enhanced signal strength on T_1_-weighted images or reduced signal strength on T_2_-weighted images 150. MRI signal intensity is governed by the T_1_ and T_2_ relaxation times of proton spins and proton spin density. Gd chelate, serving as T_1_-weighted contrast agents, demonstrate positive contrast enhancement. In contrast, superparamagnetic iron oxide nanoparticles function as T_2_-weighted MRI contrast agents, generating negative contrast enhancement 151.

Gd-based nanomaterials serve as ideal for T_1_-weighted MRI contrast agents. Multiple clinically approved Gd-based complexes incorporate single Gd ions chelated with low-molecular-weight acyclic or cyclic ligands 152. Moreover, diverse Gd-based nanoparticles and macromolecules-encompassing linear polymeric macromolecules, dendritic macromolecular nanoclusters, micelles, nanoemulsions, silica nanoparticles, protein-based carriers, gadolinium oxide nanoparticles, Gd-loaded nanotubes, and natural biological nanoparticles - have been developed as MRI contrast agents 152. For example, Luo *et al*. using BSA as a template synthesized a nanoparticle (AuGds) with multimodal imaging capabilities integrated by gold clusters and gadolinium oxide via a bio-mineralization method. The researchers evaluated the MRI capabilities of gold-gadolinium hybrids (AuGds) by comparative assessment with the clinical MRI contrast agent Gd-DTPA. The slope of the 1/T_1_ values plotted against the concentration of AuGds (12.39) is far greater than that of Gd - DTPA (3.58). On this basis, they modified FA on AuGds, and *in vivo* MRI results showed the strongest signal 4 h after injection of FA-AuGds, indicating that it could be used as the T_1_ contrast agent for MRI of tumors 153. Furthermore, Wang *et al*. engineered Au/Gd nanodots combining gold's optical/X-ray properties with Gd^3+^ spin relaxation for trimodal MRI/FL/CT imaging of bone-metastatic prostate cancer 154.

Manganese is an essential biological element. A paramagnetic ion, Mn2+ has a high spin state, a long electron spin relaxation time and fast water exchange rate. The combination of these characteristics results in a high T_1_-weighted MRI signal and bright images to provide effective positive contrast effect 155. In addition, protein coordination markedly increases the relaxivity of Mn^2+^ and gives an enhanced MRI contrast effect via the restricted molecular rotation 156. Yang *et al*. made a quantitative evaluation of the major affecting factors of T_1_-weighted MRI performance of manganese oxide nanoparticles (MnO NPs). The analysis results showed that the degree of exposure of Mn^2+^ and the specific surface area are positively correlated with T_1_ relaxivity, and the r_2_/r_1_ ratio and particle volume are negatively correlated with the sensitivity of T_1_ relaxivity. MnO octahedral nanoparticles with a high r_1_ longitudinal relaxivity value (r_1_ = 20.07 mM^-1^s^-1^) gives sensitive tumour detection with ultralow doses of 0.4 mg [Mn] per kg body weight 157. Given these advantageous properties, manganese-based nanomaterials are potential contenders for the use of T_1_-weighted MRI contrast agents.

### 5.4. Photoacoustic imaging

Based on the photoacoustic effect, photoacoustic imaging (PAI) synergizes the high contrast features of optical imaging with the deep penetration of ultrasound for a higher resolution imaging of tissue. AuNPs exhibit not only biocompatibility and chemical stability but also unique surface plasmon resonance characteristics and a high molar extinction coefficient, positioning them as leading candidates for photoacoustic imaging contrast agents 158.

For *in vivo* imaging and therapeutic agent monitoring, photoacoustic imaging is an ideal methodology. Traditional optical imaging techniques face the challenge of shallow imaging depth, whereas photoacoustic imaging can notably extend the signal penetration depth. In photoacoustic imaging, ultrasonic pressure waves generate signals through thermoelastic expansion under NIR light. Because ultrasonic waves have a much longer wavelength than light, photoacoustic signals can penetrate deeper tissues 159.

AuNPs are more stable and resistant to photobleaching than other NIR dyes. Recently, professor Gambhir's research group at Stanford University reported a miniature AuNRs that generates absorption in NIR-II, which is 5-11 times smaller than conventional AuNRs with similar aspect ratios 160. Under nanosecond pulsed laser irradiation, the thermal stability of small AuNRs and the intensity of photoacoustic signal generated by small AuNRs are 3 and 3.5 times that of large AuNRs, respectively. Theoretical and computational analyses confirm that the photoacoustic signal amplitude scales linearly with both the optical absorption coefficient of the nanoparticle suspension and the specific surface area of the nanoparticles. In tumor-bearing murine models, the small targeted nanorods enhanced tumor-specific drug delivery efficiency by 30% while generating a 4.5-fold increase in photoacoustic signal contrast.

### 5.5. Multi-mode imaging

Current mainstream medical imaging modalities-including FL imaging, CT, PET, MRI, and PAI-each possess intrinsic limitations that preclude comprehensive disease characterization when used in isolation. For instance, despite its exceptional sensitivity and real-time capabilities, fluorescence imaging suffers from restricted tissue penetration depth (< 1 mm in most biological tissues) and poor three-dimensional spatial resolution 161. CT imaging has the characteristics of high resolution, fast imaging speed and low cost 162. However, due to lack of sensitivity, CT is not suitable for identifying certain cancers or soft tissues 163. MRI has good tissue penetration but low sensitivity 164. Therefore, a multimodal imaging nanoprobe can provide more reliable information for detection and therapy.

Based on the advantages of fluorescent AuNPs, such as ultra-small size, long circulation time *in vivo*, superior light stability and low toxicity, scientists have developed various gold-based nanomaterials for multi-mode imaging 167. Zhang *et al*. used BSA-AuNCs as an imaging agent for mouse biological imaging. The results indicated that BSA-AuNCs had dual-mode imaging capabilities of fluorescence and CT 163. Liang *et al*. prepared a biocompatibility probe Gd-AuNCs for FL and MR dual-mode imaging by coupling AuNCs with Gd^3+^ 168. Yan *et al*. prepared Gd_2_O_3_-AuNCs, a multifunctional nanoplatform for near-infrared FL and MRI 169. The nanoplatform has great near-infrared FL imaging and MRI capability.

**Figure 16:**
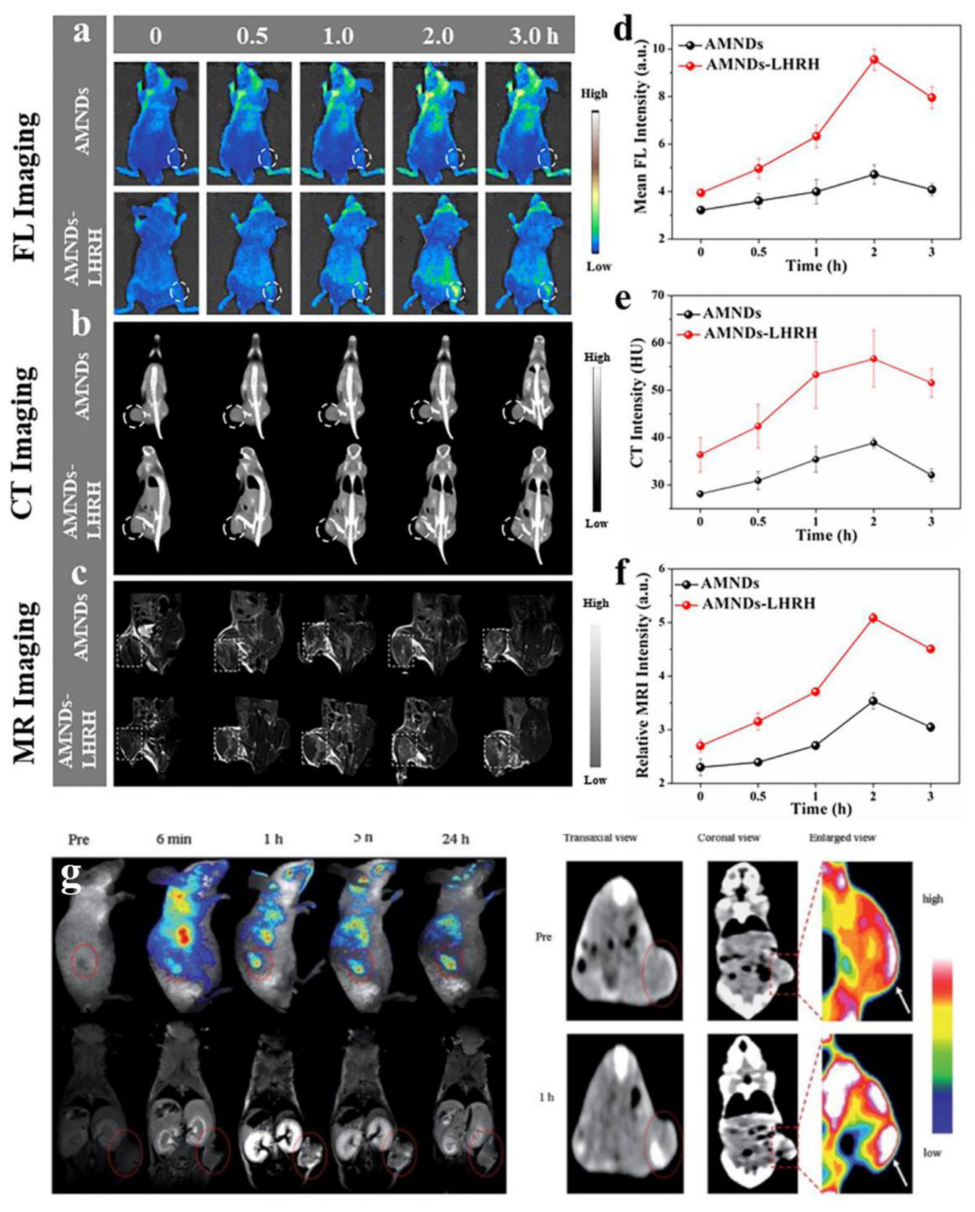
Multi-mode imaging of nanoprobe for accurate detection of tumors. (a)-(c) FL/CT/MR images of tibial prostate cancer metastasis model post intravenous injection of AMNDs and AMNDs-LHRH. (d)-(f) The FL/CT/MRI intensity of AMNDs and AMNDs-LHRH nano-system at different time points. Adapted with permission from 165. Copyright ©2023 Acta Materialia Inc. Published by Elsevier Ltd. (g) Fluorescence/MR/CT imaging of MCF-7 tumor bearing mice injection of the hybrid AuNCs. Adapted with permission from 166. Copyright © 2020 Elsevier B.V.

Recent advances have yielded protein-templated hybrid nanoprobes based on AuNCs for fluorescence/CT/MR multimodal imaging. Notably, Wang *et al*. developed a theranostic platform employing Au/Mn nanodots conjugated with luteinizing hormone-releasing hormone (AMNDs-LHRH). This nanosystem achieves integrated functions for prostate cancer management: it actively targets gonadotropin-releasing hormone receptor (GnRH-R)-overexpressing metastases to enable precision CT/MRI dual-modality preoperative diagnosis, provides intraoperative fluorescence visualization for surgical navigation, and concurrently delivers photothermal therapy. This multifunctional capability demonstrates significant translational potential spanning clinical cancer detection to guided interventions **(Figure 16a-f)** 165. Xu *et al*. used FA-functionalized BSA-AuNCs/Gd as a multi-mode imaging agent for *in vivo* targeted fluorescence, CT, and MR multimodal imaging of oral epithelial tumor models 153. Furthermore, biocompatible gold-gadolinium hybrid nanoclusters (Au-Gd NCs) stabilized by albumin matrices have been used as contrast agents for trimodal fluorescence/CT/MR imaging 168, enabling precise tumor localization through the integration of these three imaging modalities **(Figure 16g)** 170. Liu *et al*. prepared a nanoprobe SeAuFe-EpC with multi-mode imaging function. Leveraging the intrinsic X-ray attenuation capacity and strong near-infrared absorption of AuNPs, combined with the superparamagnetic property of Fe_3_O_4_, the engineered Se@Au-Fe_3_O_4_-EpCAM nanoprobe enables trimodal CT, PAI and MR imaging for *in vivo* tracking of tumor-targeting processes 171.

## 6. Application of metallic nanomaterials in tumor therapy

Traditional cancer treatments have severe side effects and limited efficacy, so a great deal of researches have turned to nanotechnology. The therapeutic landscape for intractable malignancies has been reshaped by advances in nanomaterial-mediated modalities, prominently featuring PTT, PDT, and CDT therapeutic platforms. A series of studies based on metallic nanomaterials have shifted from a single therapeutic approach to the ingenious integration of these technologies to achieve synergistic therapeutic strategies.

### 6.1. As carriers

Gold-based nanomaterials are excellent nanocarriers because of their adjustable size, excellent biocompatibility and easy surface functionalization. The combination of thiols and amines on the AuNPs surface also provides a convenient way to introduce active functional groups. Various types of drugs, targeting groups (such as antibodies, peptides), and imaging probes can be fixed to the AuNPs surface, typically by direct binding to -S or -N, or by ligand binding, electrostatic interaction adsorption, van der Waals forces, and hydrogen bonding. Compared with the strong interaction between Au and S, Au binding drugs through -N bonds usually releases drugs more effectively than through -S bonds 172. In addition, the controllable morphology of AuNPs can effectively load and release drugs. Compared with other structures, hollow Au nanoshells and Au nanocages are considered to be better drug delivery carriers 173.

As the most established cancer treatment modality, chemotherapy is widely employed clinically. Current research continues to advance our understanding of cancer pathogenesis and corresponding chemotherapeutic agents 174. Nanotechnology enables the delivery of diverse anticancer agents, enhancing therapeutic efficacy while overcoming drug resistance 175. By conjugating anticancer drugs and targeting ligands to metallic nanomaterials, tumor-specific chemotherapy can be achieved, thereby minimizing off-target effects on normal tissues. Benefiting from the superior optical properties of gold-based nanomaterials, drug release control and real-time distribution monitoring have been further promoted. For example, Wu *et al*. designed an integrated nanoplatform (with high target specificity, high payload and stimuli-responsive release) based on the superior optical properties of gold-based nanomaterials 176. They used PEG-coated Ag-Au bimetallic nanoparticle core loaded with hyaluronic acid and temozolomide (TMZ). The release of TMZ increased with the increase of temperature. The PEG-coated Ag-Au bimetallic nanoparticles have high TMZ payload, high active targeting of tumor cells, and enable intelligent drug release at specific temperatures. In addition, Yahia-Ammar *et al*. developed the Au nanocluster (120 nm) that significantly increases the amount of drug delivery and cellular uptake around tumor cells, and the drug distribution can be imaged 124.

### 6.2. Radiotherapy

**Figure 17:**
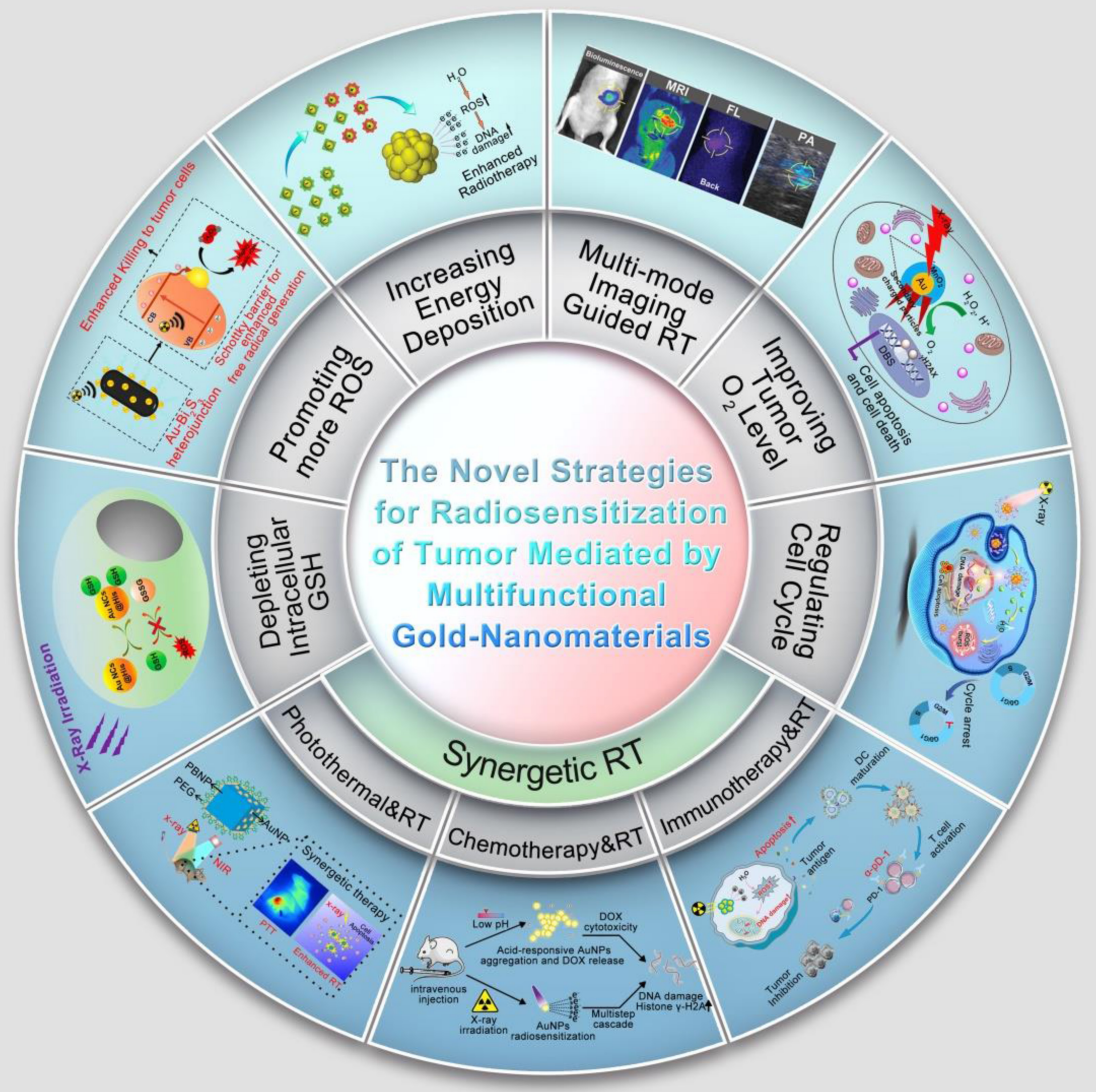
Schematic illustration of gold nanomaterial-mediated cancer radiosensitization strategies. Adapted with permission from 181. Copyright © The Royal Society of Chemistry 2023.

RT is one of the most established and effective ways of treating malignant tumors in clinical. X-ray irradiation overcomes the penetration limitation inherent to laser-based optical therapy, thus leading to a much better effect on deep-seated tumors 177. However, RT is constrained by intrinsic limitations that compromise therapeutic efficacy and long-term clinical utility 178. Currently, conventional chemotherapy drugs are used as RT sensitizers to enhance the efficacy of RT 41. However, these chemotherapy drugs are often associated with significant side effects and limited efficacy, resulting in suboptimal clinical RT outcomes 179. At the same time, the efficiency of the conversion of H_2_O to ROS did not improve. Newly developed nanomaterials hold a number of advantages for biomedicine and are actively explored for radiosensitization, such as gold-based nanomaterials. As high-Z nanomaterials, gold-based nanomaterials possess strong X-ray attenuation and can bring radiation energy deposition inside the tumours and better RT effect. Specifically, when ionizing radiation interacts with AuNPs, enhanced photon absorption occurs, leading to a localized increase in radiation dose at the tumor site. The interaction between photons and AuNPs cause a variety of effects that contribute to the emission of various types of electrons 147. These emission electrons can induce direct DNA damage or react with water to generate ROS, thus causing indirect DNA damage and ultimately leading to apoptosis 180. With the advancement of research, various novel gold-based nanomaterials have been developed to mediate tumor radiosensitization. The mechanisms underlying this radiosensitization have been elucidated, including enhanced intracellular energy deposition, promoted ROS production, regulated cell cycle progression, improved intratumoral hypoxia, depletion of GSH content in the TME, and synergistic effects when combined with other treatment modalities alongside RT **(Figure 17)**.

Targeted drugs modified in gold-based nanomaterials can be enriched in the tumor site by active targeting, and enhance their energy deposition at the tumor site after X-ray irradiation. For example, Luo *et al*. reported the gold nanomaterials (CY-PSMA-1-Au25 NCs) that targeted prostate cancer and enhanced the efficacy of RT. Experiments have shown that intravenous injection of CY-PSMA-1-Au25 NCs has high targeting to PCa, and the combination with X-ray can significantly enhance the RT effect 147.

The primary mechanism of X-ray-induced cell death involves the generation of ROS through the radiolysis of H_2_O. ROS induce cellular damage both directly, by oxidizing critical biomolecules, and indirectly, by triggering processes such as oxidative stress, apoptosis, or necrosis that ultimately lead to cell death 182. The past few years have witnessed the development of various novel multifunctional gold-based nanomaterials. These materials can generate substantial amounts of ROS under X-ray irradiation through diverse mechanisms, thereby enhancing RT efficacy. Furthermore, nanomaterials of the combination of gold and photocatalytic semiconductors such as TiO_2_ and ZnO have been fabricated specifically for RT. For example, Cheng *et al*. prepared dumbbell-shaped Au-TiO_2_ nanoparticles composed of gold and TiO_2_ nanoparticles for RT in triple-negative breast cancer (TNBC) 183. The *in vivo* experiments showed that Au-TiO_2_ nanoparticles coupled with X-ray could generate a huge amount of ROS, improve RT effect and inhibit tumour growth. In another aspect, bimetallic nano-radiosensitizers derived from Au and other precious metals like Fe or Ag can lead to the generation of a higher level of ROS through chemical catalysis reactions. Chong *et al*. constructed a new type of hyaluronic acid conjugated Au-Ag alloy nanoparticles (Au-Ag@HA NPs). Modification with HA makes the Au-Ag alloy nanoparticles take on specific targeting ability to the 4T1 breast cancer cells. Additionally, the presence of Ag endows them with better enzyme-like activities, leading to better antitumor therapeutic effects. Therefore, the enzyme-like activity of Au-Ag@HA NPs, combined with ionizing radiation, significantly promoted the generation of ROS at the tumor site, thereby significantly improving the therapeutic effect 184.

Hypoxia is a characteristic feature in the solid tumour microenvironment, which makes it resistant to RT 185. A large amount of evidence indicates that the oxygenation state of tumors largely determines the effectiveness of radiotherapy. Under normal pressure conditions during irradiation, molecular oxygen reacts with radiation-induced DNA strand breaks to form stable organic peroxides, thereby exacerbating radiation-induced DNA damage 186. Xia *et al*. developed platelet-loaded nanoparticles (Au–Hb@PLT) with gold nanoparticles and hemoglobin complex. The hemoglobin functions as oxygen carriers for alleviating hypoxic of tumours, while AuNPs act as radiosensitizers to enhance X-ray sensitivity in malignant cells. This dual functionality synergistically enhances radiotherapeutic outcomes in tumour-bearing mice under low RT dose irradiation 187. Yi *et al*. engineered Au@MnO_2_ core–shell nanoparticles with a polyethylene glycol coating, which functions as an effective radiosensitizer for improving the RT 188. Structure of Au@MnO2 core–shell structure works in two ways, the gold core promoted local X-ray energy deposition, and the MnO_2_ shell catalytically converted tumor-overexpressed H_2_O_2_ to release oxygen. This cascade rescues hypoxia resistance to radiotherapy effect in the TME.

### 6.3. PTT

PTT is an emerging oncology modality by using photothermal agent to transduce near infrared irradiation into heat within tumor areas, achieving exact tumor thermoablation. It has many advantages over the existing cancer therapies such as minimally invasive, high therapeutic efficacy, low system toxicity and controllable off-target effects 189. Hyperthermia (> 42 °C) induces irreversible biomolecular damage: enzymatic denaturation, phospholipid bilayer fragmentation, and loss of mitochondrial membrane potential, collectively driving programmed cell death 130. Although PTT comes from a variety of sources, such as radio waves, microwaves, etc., the use of lasers is more common due to its fewer side effects, low cost, and the ability to adjust the wavelength.

**Figure 18:**
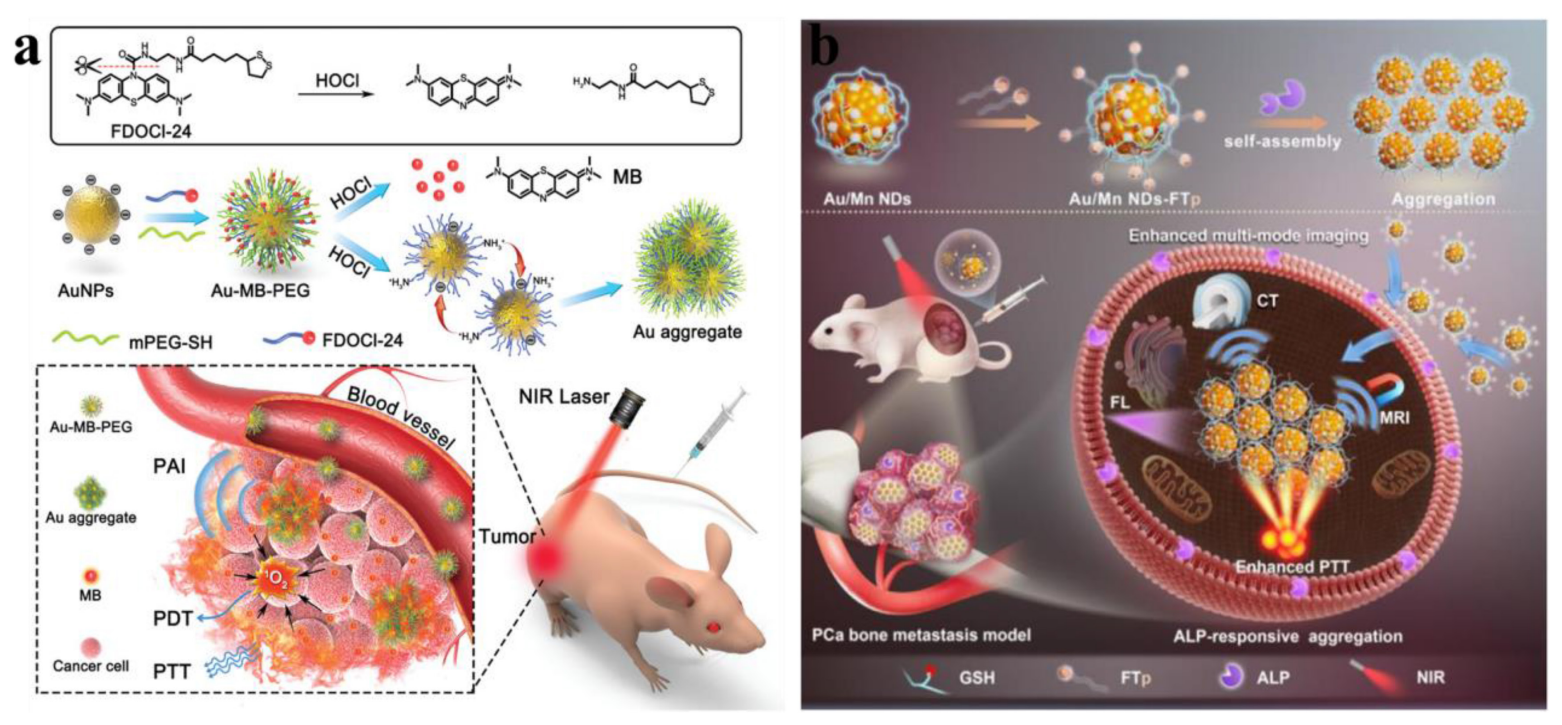
Photothermal therapy mediated by metallic nanomaterials. (a) AuNP Aggregation Activated by HOCl for PAI and Combined PTT–PDT in Cancer Treatment. Adapted with permission from 190. Copyright © 2021 The Authors. Advanced Science published by Wiley-VCH GmbH. (b) Schematic Illustration of AMNF Preparation and ALP-Induced Self-Assembly Enabling Enhanced Imaging and PTT in Metastatic Prostate Cancer. Adapted with permission from 191. Copyright ©2024 Published by Elsevier B.V. on behalf of Chinese Chemical Society and Institute of Materia Medica, Chinese Academy of Medical Sciences.

At present, the commonly used photothermal agents include coordination polyphenol polymers, metallic nanoparticles and graphene. The unique plasmonic properties of gold nanomaterials-including tunable nanodots, anisotropic AuNRs, and gold nanocages-enable high-fidelity photothermal energy conversion with minimal biological compromise, fulfilling key requirements for clinical PTT implementation. Given the size-dependent antitumor activity of nanomaterials, Yi *et al*. developed a ROS-activated gold nanoparticle aggregation strategy for image-guided PTT and PDT combination therapy **(Figure 18a)**. The ROS-activated nanoplatform Au-MB-PEG NPs are based on gold nanoparticles coated with hypochlorite probes. Au-MB-PEG NPs showed high sensitivity to hypochlorous acid, thus regulating the surface charge of AuNPs to achieve rapid accumulation, and simultaneously releasing methylene blue as a photosensitizing agent of PDT. In a tumor setting, aggregated AuNPs achieved higher tumor accumulation and retention. In addition, the aggregated AuNPs absorb redshifts, allowing them to activate PAI and PTT under near-infrared irradiation. Au-MB-PEG NPs exhibited potent antitumor efficacy *in vivo* through the synergistic action of PTT and PDT 190. The ALP-triggered self-assembling AMNF nanoplatform designed by Wang *et al*. integrates AIEgens for metastatic prostate cancer theranostics. Aggregation-induced NIR absorption augmentation drives superior photothermal performance, enabling precision tumor ablation **(Figure 18b)** 191.

Li's team prepared a macrophage membrane coated gold nanomaterial (MPCM-AuNS) for PTT *in vivo*. Macrophage membrane coating endows nanoparticles with the ability of active targeting, and after intravenous injection of MPCM-AuNS, its circulation time *in vivo* is prolonged and its accumulation at the tumor site is enhanced 192. The spatially controlled hyperthermia generated by MPCM-AuNS during NIR irradiation enables precision oncotherapy through simultaneous tumor growth inhibition and targeted cell ablation. This biomimetic strategy exemplifies gold nanomaterial optimization for PTT. Li's Au@Ag core-shell nanoparticles further advancing the field via enhanced photothermal transduction in the biological window 193. The superior photothermal transduction ability of Au-Ag core-shell nanostructures induce hyperthermia under laser irradiation. Reports *in vitro* showed death rate of cells in different time scales, and total tumor cell death could be reached after short-time irradiation.

Substantial experimental evidence has verified that the synergic effect of bimetallic photothermal nanoagents is superior to their monometallic counterparts in photothermal energy transformation on the synergistic electronic effects at the metal interfaces. For example, Tang *et al*. demonstrated that Au@Pt nanostructures (AuNRs coated with Pt nanodots shells) have better photothermal effects than AuNRs 194. At the same laser fluence, Au@Pt nanostructures have faster thermal dynamics and higher temperature than Au nanorods, which generate strong cytotoxicity of tumor cells. Meanwhile, Au@Pt nanostructures presented superior PTT effect than Au nanorods in a mouse tumor model. This is also studied by another hollow Au-Ag alloy nanomaterial (HAAA-NUs) 195. HAAA-NUs are shaped like sea urchin and its photothermal conversion ability is superior to that of Au colloids. HAAA-NUs have good biocompatibility, but have laser-induced cytotoxicity. The therapeutic effect is positively correlated with the laser power. After injection of HAAA-NUs and laser irradiation, the tumor volume decreased and finally was totally eliminated. Chen *et al*. used polyethylene glycol modified Au@Pt nanoparticles loaded with cerium dioxide (CeO_2_/Au@Pt-PEG) to achieve superior PTT targeting effect 196. CeO_2_/Au@Pt-PEG exhibited superior photothermal properties and achieved marked antitumor efficacy in both cellular and murine models.

### 6.4. Synergistic therapy

**Figure 19:**
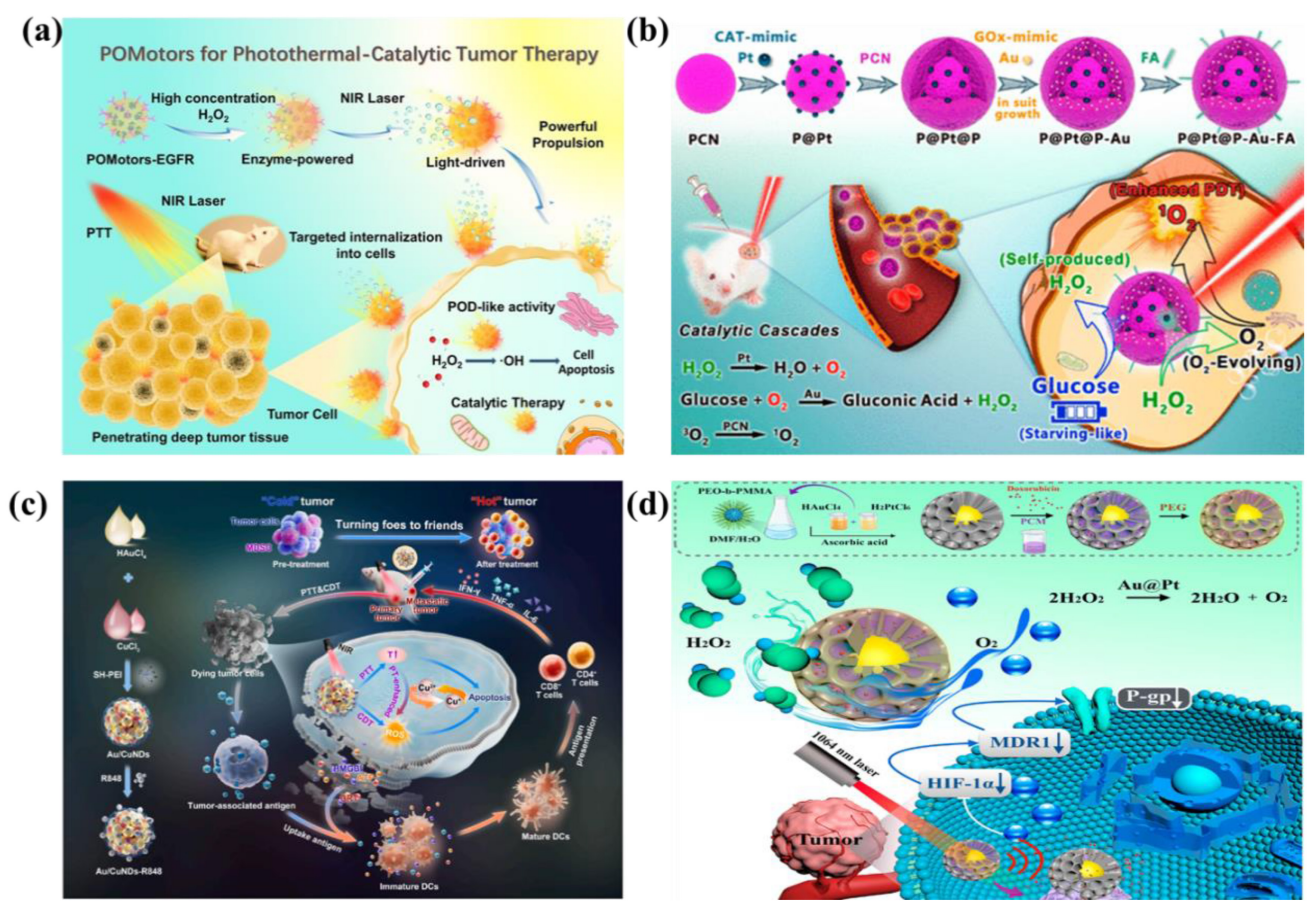
The synergistic therapy mediated by metallic nanomaterials for tumor treatment. (a) Schematic of self-propelled POM-based nanomotors enabling photothermal-catalytic synergistic therapy. Adapted with permission from 114. Copyright © 2023 Wiley-VCH GmbH. (b) Dual inorganic nanozyme-engineered porphyrin MOFs for catalytic cascade-enhanced therapy. Adapted with permission from 197. Copyright © 2019, American Chemical Society. (c) Au/CuNDs-R848 *in situ* nanovaccine for dual-mode imaging-guided therapy with dual immunomodulation against metastatic TNBC. Adapted with permission from 198. Copyright © 2024 The Authors. Publishing services by Elsevier B.V. on behalf of KeAi Communications Co. Ltd. (d) Synthetic route and therapeutic mechanism of multifunctional Au@Pt-DOX-PCM-PEG nanotherapeutics. Adapted with permission from 199. Copyright © 2022 American Chemical Society.

Emerging nanotherapeutics offers an unprecedented solution to the replacement of surgeries. More and more evidence has proved that combination therapy outperform monotherapeutic therapy, for example, PTT or PDT therapy with other treatments. Metallic nanomaterials can achieve the combined manner from their excellent photonic properties. An example of this progress is the light-driven nanomotor based on polyoxometalate nanozymes for targeted tumor therapy in photothermal-catalytic synergy 114. Within this architecture, the POD-like activity of P_2_W_18_Fe_4_-based nanomotors gives autonomous propulsion to increase the ROS production for attacking the tumour cell. The synchronous addition of the polydopamine for conjugation offers the self-propelling function. Following 10-minute irradiation (808 nm), epidermal growth factor receptor antibodies guide tumor-targeted accumulation and penetration of the nanomotors, achieving efficient photothermal-catalytic synergy **(Figure 19a)**. Liu *et al*. established the bimetallic nanomaterials with two kinds of enzyme-like activity, and realized the combined treatment of PDT and starvation therapy 197. As shown in **Figure 19b**, the porphyrin metal-organic framework was used as a carrier to incorporate PtNPs with CAT-like function and AuNPs with GOx-like function. Finally, FA is grafted onto the surface of the nanomaterial to obtain P@Pt@P-Au-FA. The dual-nanozyme platform converts glucose into H_2_O_2_, subsequently driving the generation of oxygen. The elevated O₂ levels not only enhance glucose consumption, contributing to effective starvation therapy, but also boost the production of reactive ROS during PDT. When used in conjunction with laser irradiation, P@Pt@P-Au-FA displays remarkable therapeutic efficacy.

In recent years, immunotherapy has developed rapidly and could achieve better results when combined with other treatment methods. In our group, we designed an "*in situ* nanovaccine" Au/CuNDs-R848 for image-guided PTT/CDT synergistic therapy to trigger dual immunomodulatory effect on TNBC 198. As a dual-functional photothermal agent and nanozyme, Au/CuNDs-R848 enable PTT and CDT against primary TNBC tumors. The concurrently released TAAs and DAMPs stimulate dendritic cell maturation and potentiate cytotoxic T lymphocyte infiltration. Consequently, Au/CuNDs-R848 function as an *in situ* nanovaccine that enhance TNBC immunogenicity through immunogenic cell death. Through such dual immunomodulation-converting immunologically "cold" tumors into "hot" phenotypes-it implements a tumor microenvironment remodeling strategy while augmenting metastatic TNBC immunotherapy. Additionally, Au/CuNDs-R848 serve as a multimodal nanoprobe, facilitating high-resolution NIR FL and CT for precise TNBC visualization **(Figure 19c)**. Sun *et al*. reported on nanotherapeutic drugs Au@Pt-DOX-PCM-PEG combining chemotherapy, PTT, and gene therapy **(Figure 19d)** 199. Au@Pt-DOX-PCM-PEG alleviates tumor hypoxia, reduces tumor heat tolerance and improves PTT treatment efficiency through gene therapy. In addition, genic-enhanced PTT combined with chemotherapy can significantly enhance the efficacy of tumor treatment.

**Figure 20:**
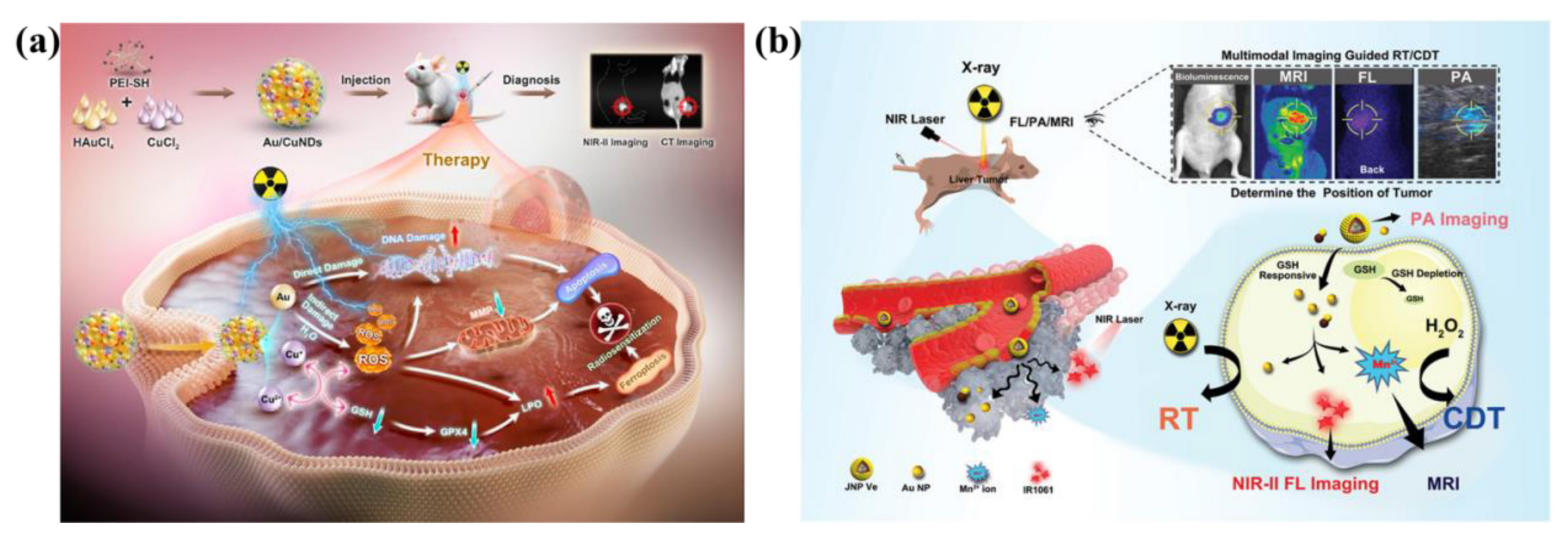
Integration of tumor diagnosis and treatment mediated by metallic nanomaterials. (a) Depiction of Au/CuNDs synthesis for usage in dual-mode imaging and apoptosis/ferroptosis-mediated high-efficiency radiosensitization. Adapted with permission from 66. Copyright © 2024 American Chemical Society. (b) Schematic illustration of image-guided RT against deep-seated tumors. Adapted with permission from 200. Copyright © 2021 Wiley-VCH GmbH.

Metallic nanomaterials have excellent multi-mode imaging effect. To address the demand for integrated theranostics combining collaborative therapy with multimode imaging, Au/Cu nanodots (Au/CuNDs) were developed. This platform enables dual-mode imaging, amplified ROS production, and induction of apoptosis-ferroptosis for effective radiosensitization 66. Au/CuNDs exhibit potent radiosensitization, POD-like activity, and GSH-depleting capability, thereby generating substantial ROS. This ROS, synergizing with ionizing radiation, induces apoptosis via DNA damage amplification and ferroptosis through lipid peroxidation enhancement, significantly enhancing RT efficacy. Furthermore, Au/CuNDs function as a multimodal nanoprobe, enabling high-resolution NIR FL imaging and CT for tumor visualization **(Figure 20a)**. Lin *et al*. designed a GSH-responsive nanovesicles loaded with near-infrared fluorescent dye and gold-manganese nanoparticles for multimodal image-guided RT and CDT synergistic tumor therapy **(Figure 20b)**. The location of the tumor can be accurately located through multi-mode imaging to achieve image-guided precision treatment and minimize the side effects on healthy tissues 200. Multi-modal cancer therapy utilizing metal-based nanomaterials integrates the benefits of diverse treatment modalities to achieve superior therapeutic outcomes, heralding a new paradigm of synergistic cancer therapy.

## 7. Future directions and conclusions

Although researchers have made some progress in the design and preparation of multifunctional gold-based nanomaterials, the potential application in the biomedical field and further optimization of the design still need to be further explored. At the end of this paper, the prospect of related work is presented:

Firstly, bimetallic nanomaterials-comprising two distinct metallic elements-exhibit enhanced functional characteristics compared to monometallic counterparts. These materials have garnered extensive research attention and translational applications in biomedicine, particularly for cancer therapy, owing to their unique morphological architectures, distinctive physicochemical properties, superior biocompatibility, and inherent synergistic effects. However, how to further optimize the preparation methods of bimetallic nanomaterials, improve their performance, and fully leverage the advantages of bimetallic nanomaterials in tumor treatment still requires further exploration.

Secondly, metal nanozymes, as artificial mimetic enzymes, have become a research hotspot in the field of tumor diagnosis and treatment due to their unique catalytic performance, high stability and designability. Metal nanozymes can effectively address the limitations of natural enzymes, such as poor stability, complex preparation and high price. The development of novel and efficient metal nanozymes, such as cascade catalytic systems and single/diatomic nanozymes, represents a key of future research. As research advances, metal nanozymes-mediated nanomotors are a novel direction, which can promote their diffusion to the deep part of the tumor and escape of lysosomes by generating self-dynamics, hereby improving diagnostic and therapeutic efficacy.

Thirdly, due to the unique physical properties of metal-based nanomaterials, they have become important milestones in the clinical translation of radiosensitization (such as NBTXR3, AGuIX, CYT-6091, etc.), and some nanomaterials have entered phase 3 clinical trials. Admittedly, this paper systematically explores the phenomenon of radiation sensitization effect of metallic nanomaterials. However, the current mechanism research is still mainly limited to the positive verification of protein phenotypes and related molecular pathway levels. Further rescue experiments are needed to more rigorously confirm the regulatory correlation between molecular pathways.

Fourth, laboratory-level synthesis and industrial production follow different chemical engineering principles, and many efficient synthetic pathways are difficult to directly scale up linearly. In the future, it is urgently necessary to enhance interdisciplinary cooperation between chemists and engineers, focusing on the definition and optimization of key process parameters, to achieve stable, controllable and large-scale preparation of nanomaterials, and ensure that their key quality attributes are highly consistent with those of laboratory samples.

Fifth, at present, there is still a lack of unified standards for the biocompatibility assessment of metallic nanomaterials, and the research of metabolic processes is also not rigorous enough *in vivo*. Subsequently, radioactive isotope labeling or elemental analysis techniques can be utilized to precisely map the spatiotemporal distribution of nanomaterials in normal and tumor-bearing animal models, clarify the pharmacokinetic characteristics, and quantitatively track their metabolic and excretory pathways, thereby obtaining clear absorption, distribution, metabolism, and excretion data.

Sixth, for a long time, researches have mostly focused on the functions of nanomaterials themselves, while there were insufficient understanding of their complex interactions with the host immune system. Metallic nanomaterials not only can activate the immune system but also induce immunosuppression. This is a significant dual effect, which is closely related to the physicochemical properties of metallic nanomaterials and the microenvironment. In the future, the remodelling effect of nanomaterials on tumour immune microenvironment needs to be paid more attention. With the combination of flow cytometry and single-cell sequencing techniques, we could systematically analyse the quantitative and functional changes of key immune cell subsets in the tumour microenvironment, such as tumour-infiltrating CD8^+^ T cells, suppressive T and myelogenic suppressor cells, which will be significant for the use of metallic nanomaterials in immunotherapy.

Finally, metal-based nanomaterials are at a critical juncture in the transformation process from laboratory concepts to clinical drugs. The breakthrough in the future does not lie in synthesizing more complex materials, but in whether we can confront and solve the core issues raised above. Only by bridging these gaps from phenomenon to essence can the ultimate vision of its clinical transformation be realized.

## Figures and Tables

**Table 1:** Comparative Analysis of AuNPs Synthesis Methods.

Method	Advantages	Limitations	Key Performance Characteristics
Physical(γ-ray/UV/laser irradiation)	• High purity• Precise size control• Reproducible results	• High energy consumption• Specialized equipment required	• Tunable optical properties• Narrow size distribution (< 5 nm achievable via laser)
Chemical(Reduction/electrochemical)	• Rapid synthesis• High yield control• Cost-effective scalability	• Toxic reagents • Residual stabilizer contamination	• Superior crystallinity• Size modulation via surfactants/temperature
Biological(Plant/microbial synthesis)	• Eco-friendly• Non-toxic byproducts• Inherent biocompatibility	• Complex purification• Batch-to-batch variability• Slow reaction kinetics	• Enhanced biocompatibility for biomedicine• Enzyme-mediated size reduction
Electrochemical	• High particle quality• Precise current-density control• Scalable production	• Limited shape diversity• Electrode maintenance required	• Superior monodispersity• Rapid synthesis speed
